# Predicting the spread of covid-19 and the impact of government measures at the early stage of the pandemic: The Dutch case—Stricter but short-term measures are better

**DOI:** 10.1371/journal.pone.0283086

**Published:** 2023-05-12

**Authors:** Cyrelle J. Tenhagen, Engin Topan, Karin C. G. M. Groothuis-Oudshoorn

**Affiliations:** 1 Industrial Engineering and Business Information Systems, Faculty of Behavioural Management and Social Sciences, University of Twente, Enschede, The Netherlands; 2 Health Technology and Services Research, Faculty of Behavioural Management and Social Sciences, University of Twente, Enschede, The Netherlands; Jeonbuk National University, REPUBLIC OF KOREA

## Abstract

In this paper, we investigate the spread of COVID-19 and the impact of government measures at the early stage of the pandemic (before the introduction of the vaccines) in the Netherlands. We build a multiple linear regression model to predict the effective reproduction rate using key factors and measures and integrate it with a system dynamics model to predict the spread and the impact of measures against COVID-19. Data from February to November 2020 is used to train the model and data until December 2020 is used to validate the model. We use data about the *key factors*, e.g., disease specific such as basic reproduction rate and incubation period, weather related factors such as temperature, and controllable factors such as testing capacity. We consider particularly the following *measures* taken by the government: wearing facemasks, event allowance, school closure, catering services closure, and self-quarantine. Studying the strategy of the Dutch government, we control these measures by following four main policies: doing nothing, mitigation, curbing, elimination. We develop a systems dynamic model to simulate the effect of policies. Based on our numerical experiments, we develop the following main insights: It is more effective to implement strict, sharp measures earlier but for a shorter duration than to introduce measures gradually for a longer duration. This way, we can prevent a quick rise in the number of infected cases but also to reduce the number of days under measures. Combining the measures with a high testing capacity and with effective self-quarantine can significantly reduce the spread of COVID-19.

## 1. Introduction

The COVID-19 pandemic has affected the social and economic life of the world severely. Governments all over the world have taken several measures to control the pandemic. Yet, governments still do not exactly know how much impact measures have on limiting the spread of the pandemic, and how other factors (e.g. weather) impact the spread exactly. Furthermore, each country has different demographics and circumstances, which makes the effectiveness of measures differ between countries [1, 2]. In this paper, we investigate the spread of COVID-19 and the impact of government measures in the Netherlands.

According to Red Team, an independent group of experts that aims to prevent and fight COVID-19 in the Netherlands, there exist roughly four strategies to determine the policy that can prevent a virus from spreading. These are “Do nothing”, “Mitigation”, “Curbing”, and “Elimination”. *Do nothing* does not take action to prevent spread and the virus can move freely. *Mitigation* allows circulation of the virus to a certain extent and measures are put in place to avoid overuse of hospital and IC capacity. *Curbing* strives for as little infections as possible, pursues every infection. *Elimination* aims to make the virus disappear. The policy that was chosen by the Dutch government is called “maximal control”, which can be seen as a mix of policies mitigation and curbing. The main focus of the government is to protect people who are vulnerable and prevent healthcare from overloading [3].

To predict the spread of the pandemic and analyze impact of measures, we use a two-step approach. In the first step, we use a *multiple linear regression (MLR)* model to explain the relation between the spread, the key factors and government measures. The *key factors* that we consider are: disease related: incubation period, infectious period, basic reproduction rate, fatality ratio, initial infected cases; weather related: temperature, humidity, wind speed; and controllable factors: adoption of government measures and testing. The government *measures* that we consider are event allowance, school closure, catering services closure, wearing facemasks, and self-quarantine. In the second step, we bring the key factors and measures in a simulation environment using a *System Dynamics (SD)* model. We use system dynamics to model the spread of COVID-19 of the early phase of the pandemic, with which we can represent the structure of a complex system and analyze dynamic behaviour over time [4]. In the SD model we use the *effective reproduction rate* as an indicator of the spread. This rate is influenced by external factors like government measures [5]. Since SD uses a high level of aggregation, we combine our SD model with MRL to enhance predictions. The SD model can capture the dynamic nature of the pandemic and MLR incorporates the more detailed hidden relations between factors and the spread of the pandemic.

We study the spread of COVID-19 in the Netherlands and use the data publicly available until 31 December 2020. We build and calibrate the SD model predicting future spread of the pandemic using data until 1 December 2020. With this model we develop insights for policymaking by considering different policies to prevent spread, discover the impact of timing and strictness of measures, and test whether testing capacity affects the effectiveness of a policy in the Dutch case.

The remainder of this paper is organized as follows: In Section 2, we discuss the contribution to the literature. In Section 3, we introduce our data analysis methodology and develop the prediction model. Numerical implementation and results are presented in Section 4, and in Section 5 we conclude main findings and provide the foundation for further work.

## 2. Contribution to the literature

There have been several research papers on estimating the individual impact of factors such as incubation period and weather, and also on the impact of government measures such as school closure on the spread of COVID-19 [6–15]. But since there exists no magical measure that is able to decrease the effective reproduction rate below one on its own, a suitable combination of measures is necessary to effectively prevent spread of the virus. Next to the combination of measures, timing of the implementation of these measures can drastically influence the impact [16, 17]. And combining effective measures with a sufficient testing policy is considered to be important to end the pandemic [18, 19]. In addition there are several other country specific factors influencing the spread of COVID-19 such as population density and the acceptance of implemented measures by the public in that specific country. This indicates that each country can require a different policy to work best. Yet studies on effectiveness of measures in the Netherlands either look at the general impact instead of the country specific impact, or determine the effectiveness of a particular measure. This paper takes a broader perspective. We investigate the impact of all measures taken by the government, considering other aspects such as weather related factors, using in a dynamic model.

Various SD models are developed to predict spread of COVID-19 [20–25]. Combining SD modelling with other methods allows to enhance the contribution of modeling work and develop potential solutions in more profound ways than a single-method study can do [23, 24]. Yet, none of these models enhances predictors by incorporating MRL. While MRL shows to be an effective method to predict the spread of COVID-19 [26–28]. We contribute to the literature by developing a method combining (i) SD to capture the dynamic nature of the pandemics and (ii) MRL to integrate it with detailed relations between factors and response variables. In this way we can integrate the effectiveness of policies to prevent the spread of COVID-19 in the Netherlands that include the effect of testing and the timing of the implementation of measures in the Dutch case.

## 3. Methodology

This section introduces our method. First we introduce our SD model in Section 3.1 and the MLR model in Section 3.2. In Section 3.3, we calibrate the SD model integrated with the MLR model. And in Section 3.4 we explain how we model all policies (doing nothing, mitigation, curbing, and elimination).

### 3.1 The system dynamics model

There are a number of well-known classical *epidemiological models* that can express outbreak dynamics in the SD model. We apply an extension of the SIR compartment model from Kermack and McKendick [29]. In the SIR model, individuals pass through different stages as they experience the disease. The SIR model distinguishes susceptible, infected, and recovered compartments in the population. In the extension we apply, the *SEIR model*, an exposed compartment is included additionally. This is useful due to the relatively long latency phase of COVID-19.

#### 3.1.1 Dynamics of the SD model

**Fig 1:**
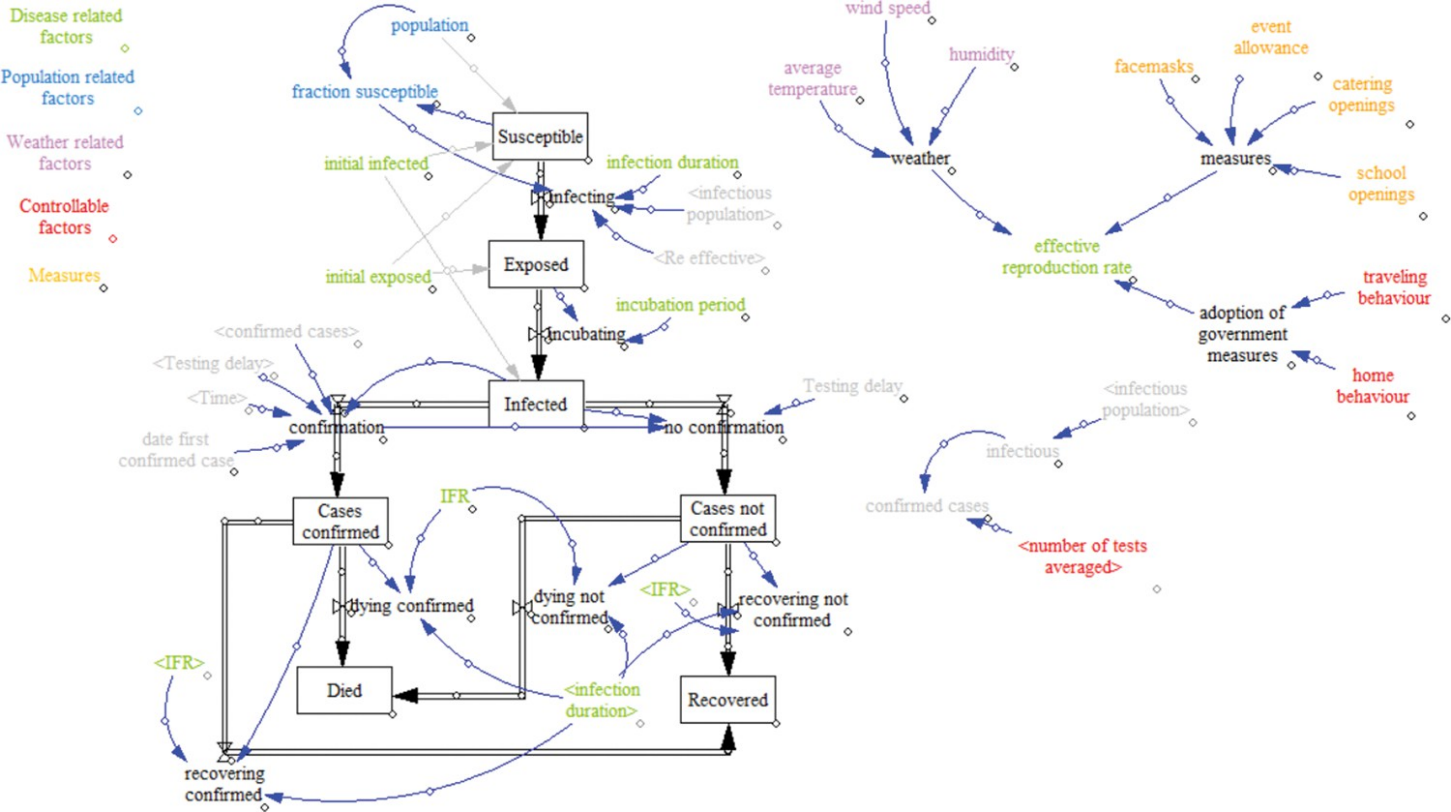
The SD model.


With our SD model, visualized in Fig 1, we express the spread of COVID-19 in the Netherlands. Quantities of the compartments (susceptible, exposed, infected, recovered) in the SD model are changing over time and can be mathematically formulated with differential equations. Below we provide the differential equations indicating the change in susceptible, exposed, infected, and recovered population per day, expressed with $\frac{dS}{dt},\frac{dE}{dt},\frac{dI}{dt}$ and $\frac{dR}{dt}$ respectively.

$$\frac{dS}{dt}=-\beta SI$$

$$\frac{dE}{dt}=\beta SI-\propto E$$

$$\frac{dI}{dt}=\propto E-\gamma I$$

$$\frac{dR}{dt}=\gamma I$$

Fractions *S*, *E*, *I*, and *R* represent the fraction of susceptible, infected, and recovered individuals respectively. The four population fractions resemble the entire Dutch population, meaning *S*+*E*+*I*+*R* = 1. Additionally, we distinguish infected population in confirmed and unconfirmed with help of a regression model for the number of confirmed cases (see Section 3.2.3). Similarly, we distinguish recovered population in recovered and deceased using *Infection Fatality Ratio* (IFR).

#### 3.1.2 Input for the SD model

Being nonlinear models, SD models are sensitive to input parameters, which requires rigorous parameter estimation [30]. To determine the effect of each parameter on the spread, we use multiple linear regression. In our study, we develop two regression models. In the first regression model, we take the effective reproduction rate (*R*_*e*_(*t*)) as a response and explain the relation between effective reproduction rate and factors and measures. The second regression model expresses the number of confirmed cases per day with respect to the input variable testing capacity.

We identify five groups of input parameters in our SD model that affect the spread of COVID-19. These include *disease related uncontrollable factors*, *population related uncontrollable factors*, *weather related uncontrollable factors*, *controllable factors* and *government measures*.

*Disease related factors affecting the spread of COVID-19*. Disease related uncontrollable factors include incubation period, infectious period, basic reproduction rate, fatality ratio and initial infected cases. Ranges of the disease related factors are provided below. We calibrate input values of these factors to mimic actual spread (Section 3.3).

- *Incubation period*: The time between infection and symptom onset. This period is estimated to be on the average five to six days, but can be between 2 to 14 days [31].
- *Infectious period*: The period an infected person is able to infect another person. This period is estimated to start approximately two days before symptom onset with a range between one and five days [31]. The proportion of transmissions before symptom onset was estimated to be 44%, yet the accuracy of this estimate has to be questioned due to a lack of data. The infectious period continues for up to seven days from the onset of symptoms with a peak at 0.7 days [32].
- *Basic reproduction rate (R*_0_): This rate indicates how many people are infected by one infected person on average. The basic reproduction rate applies when no outbreak control is applied to limit the spread, and defines the rate as the average number of cases produced by an infected individual in a fully susceptible population [33]. The RIVM (the Dutch National Institute for Public Health and Environment) estimates *R*_0_ to be between 2 and 2.5 [34].
- *Infection fatality ratio*: The number of deaths divided by the number of infections. We use the IFR to indicate the number of deaths after infection. The IFR ranges between 0.3% and 1% [7].

*Population related factors affecting the spread of COVID-19*. Population related uncontrollable factors include the population size and the fraction of susceptible cases. The population size of the Netherlands is assumed to be fixed at 17,400,000 on the first day of simulation. The fraction of susceptible cases depends on the number of *initial infected cases*.

*Weather related uncontrollable factors affecting the spread of COVID-19*. We express weather with wind speed, humidity, and temperature. We gather values from the KNMI (the Royal Netherlands Meteorological Institute) measured in the Bilt, a municipality in the middle of the Netherlands [35]. We use these values to represent values everywhere in the Netherlands.

*Controllable factors affecting the spread of COVID-19*. We identify adoption of government measures, testing capacity, and self-quarantine as controllable factors that might affect the spread.

**Fig 2:**
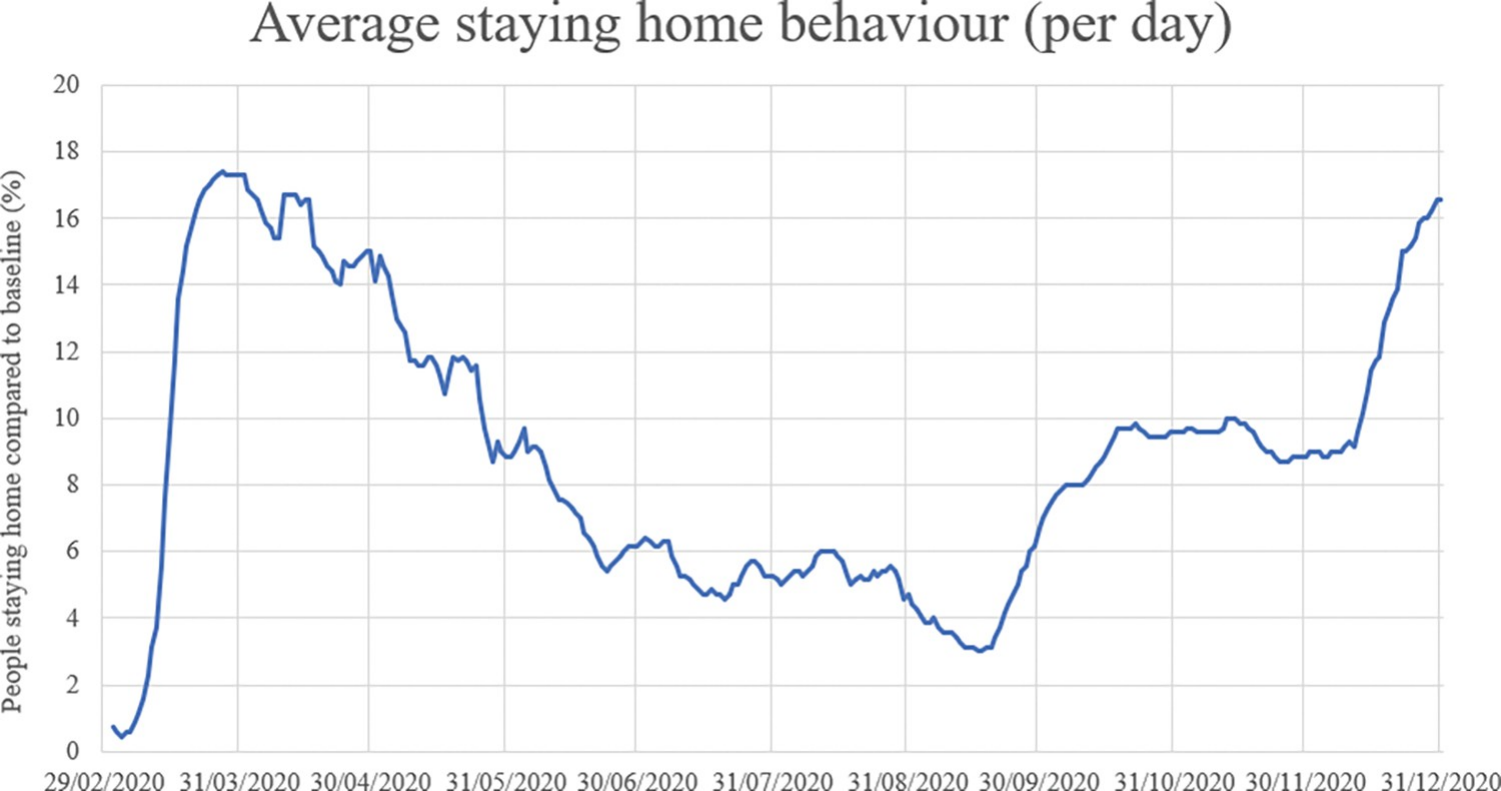
Development of the staying home behaviour in the Netherlands.

**Fig 3:**
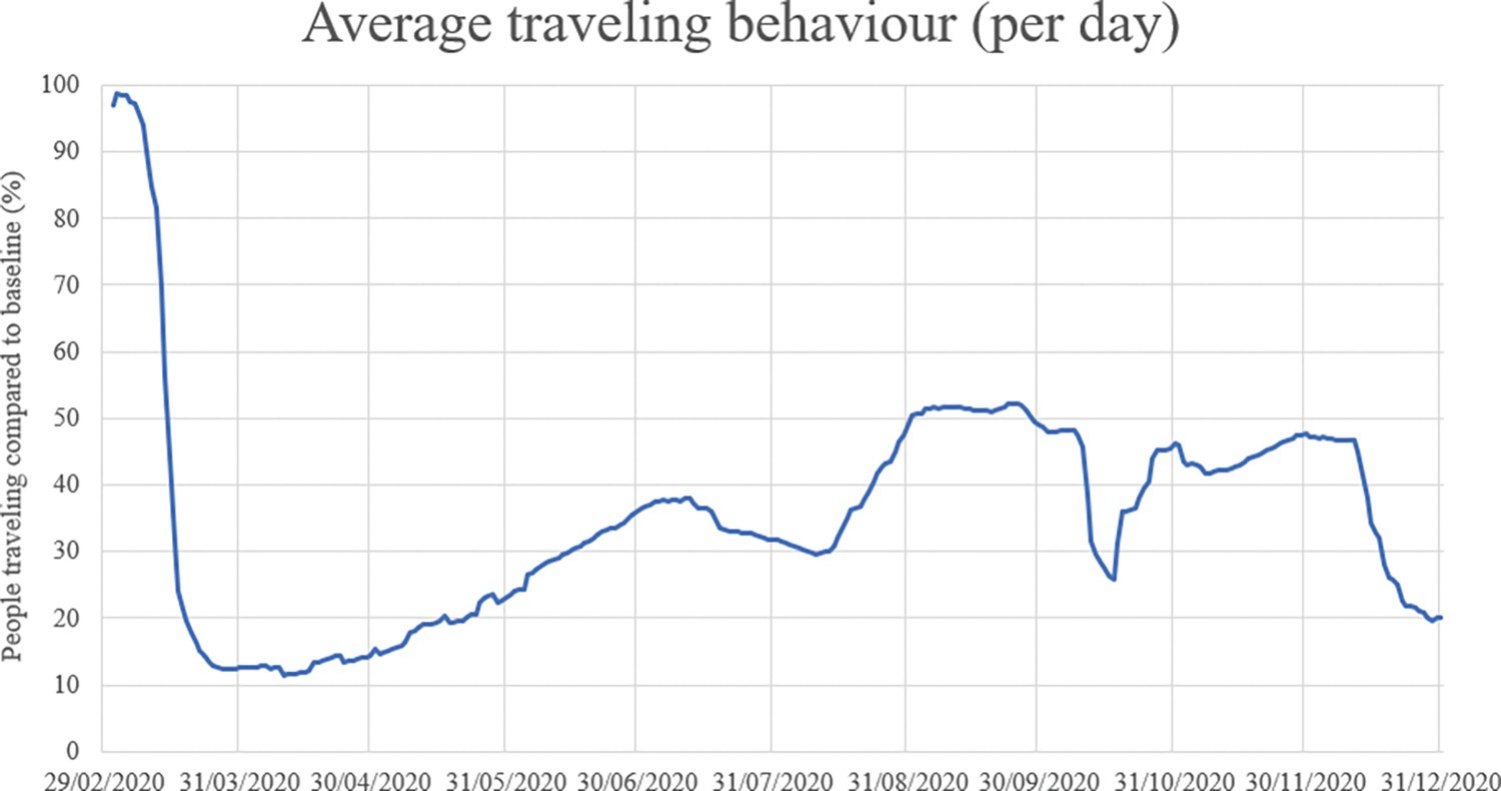
Development of the travelling behaviour in the Netherlands.

**Fig 4:**
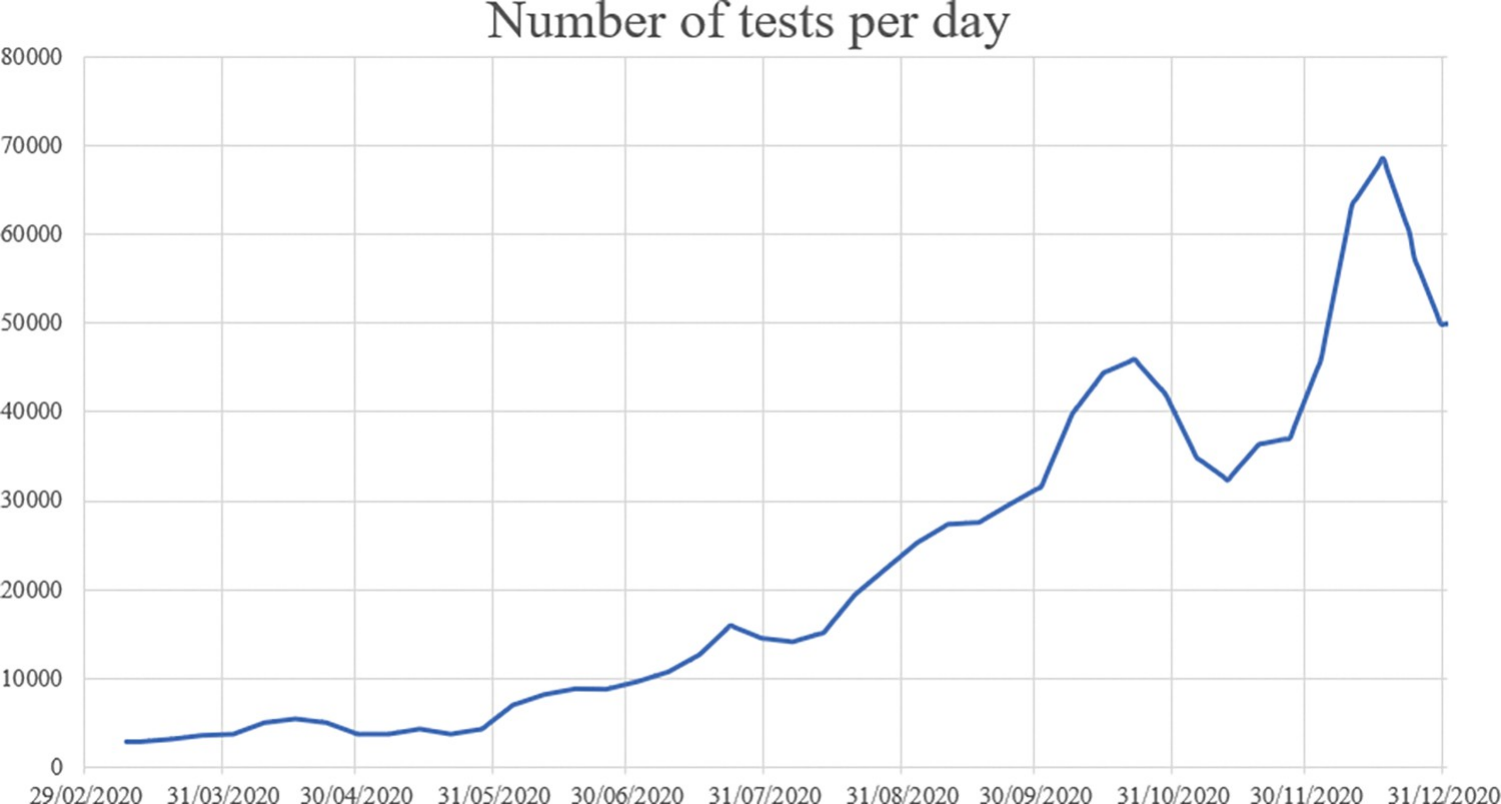
Development of the number of tests per day in the Netherlands.

- *Adoption of government measures*: We use the number of people staying home per day (“*staying at home behaviour*”) and the extent by which people travel with public transport per day (“*traveling behaviour*”) to express adoption of government measures. We quantify staying home behaviour with Google data about how often people stay at home per day compared to a baseline, and we quantify traveling behaviour with data about public transport gathered from Translink [36, 37]. Staying home behaviour and traveling behaviour per day are visualized in Figs 2 and 3 respectively.

- *Testing capacity*: We express testing capacity with the number of tests per day, visualized in Fig 4. Data before 1 June is gathered from Datagraver and data after 1 June from the Dutch Central Government (“Rijksoverheid”) [38, 39].
- *Self-quarantine*: Besides including the effect of the places of infection above with help of measures, we include the effect of self-quarantine in our model. This measure is included with a fixed value by defining the fraction of infected cases who are effectively quarantined. According to data from the RIVM, approximately 50% of reported infection places is at home [40]. The effectivity of self-quarantine is therefore estimated to be between 50 and 70%.

### Government measures affecting the spread of COVID-19

The measures taken by the Dutch government are mostly implemented to reduce the contact rate in certain places of infection. However, schools, catering services, and events and gatherings are places where a considerable number of infections can occur, and are thus key for the effectiveness of government measures [40]. Therefore, we include them explicitly in our model. The degree by which schools and catering services are opened, events are allowed, and facemasks are implemented is quantified per day based on the definition of measures in Table 1 (referred to as school closure, catering service closure, event allowances, and facemasks respectively). For example, when all schools are closed, we consider the measure to be very strict which we indicate with a value of 5. Historical values of these measures are clarified in the S1 Table, with additional dates of implementation.

**Table 1:** Quantification of government measures.

**Measure**	**Value**	**Definition**
*Schools closure*	1	Normal education. Fully physical.
	2	Mostly physical education / Most schools open.
	3	Partially physical, partially non-physical education / Approx. half of schools opened.
	4	Mostly non-physical education / most schools closed.
	5	All schools closed / only online education.
*Catering service closure*	1	All catering services normally opened.
	2	All catering services normally opened with distancing measures.
	3	All catering services opened, closing at 12 PM. With distancing measures
	4	All catering services opened, closing at 10 PM. With distancing measures
	5	All catering services closed.
*Events allowance*	1	All events allowed.
	2	Some events cancelled.
	3	Big events prohibited.
	4	Events prohibited and gatherings with maximum number of people.
	5	All events and gatherings are prohibited. No maximum
*Facemasks*	1	No facemasks.
	2	Facemasks in public transport only.
	3	Urge to wear facemasks.
	4	Strong urge to wear facemasks in all public spaces.
	5	Facemasks mandatory in all public spaces.

### 3.2 Multiple linear regression models for key indicators

We use two linear regression models. The first one expresses the effective reproduction rate in terms of weather-related factors, controllable factors and measures (Section 3.2.2). The second one expresses the relationship between the number of confirmed cases on a day and the test capacity (Section 3.2.3). We apply multiple linear regression to identify relations between parameters and to determine whether weather-related factors, controllable factors and measures have a considerable influence on the spread.

#### 3.2.1 Performance measures of the multiple linear regression model

We use *R*^2^, the adjusted *R*^2^ and the test error to assess the performance of our regression model. We express the test error with the mean squared error (MSE). To avoid an optimistic outcome of the performance measures, we correct *R*^2^ and MSE for optimism by performing bootstrap. We call the resulting performance measures “corrected *R*^2^” and “corrected MSE”. We use the bootstrap method to estimate the accuracy of the method by running the method multiple (500) times, each time with a different sample set [41].

#### 3.2.2 Multiple linear regression model for effective reproduction rate

*Effective reproduction rate*. The effective reproduction rate *R*_*e*_(*t*) is influenced by external factors like government measures. To incorporate impact of changing measures and factors, we develop a regression model that can express *R*_*e*_(*t*) of COVID-19 in the Netherlands as a function of these factors and measures on any day *t*.

Before we can identify the effect of controllable factors and measures on *R*_*e*_(*t*) we have to calculate *R*_*e*_(*t*), to be able to compare the outcome of our model to the actual reproduction rate when making predictions in the future. We refer to the calculated effective reproduction rate as *R*_*e*_^*calculated*^(*t*). We calculate *R*_*e*_^*calculated*^(*t*) in R with an implementation of the method provided by Wallinga & Teunis [42]. The method estimates the time dependent reproduction number together with a confidence interval. We assume a gamma-distribution for the generation interval with a mean of 4 days and a standard deviation of 3 days in this function [43, 44]. A visualization of *R*_*e*_^*calculated*^(*t*), from 20 February until 31 November, is provided in the S1 Fig, based on the number of infections and the number of hospitalizations respectively.

### Regression model for the effective reproduction rate

We apply the backward selection method to determine the relevance of weather related factors, controllable factors and government measures and thus to determine whether a considerable relation exists [45]. This provides the following model for *R*_*e*_(*t*):

$$R_{e}(t)=1.259-0.014*average\;temperature-2.669*staying\;home\;behaviour+0.919*traveling\;behaviour+0.047*school\;closure-0.041*catering\;service\;closure-0.076*event\;allowance+0.061*facemasks$$

**Fig 5:**
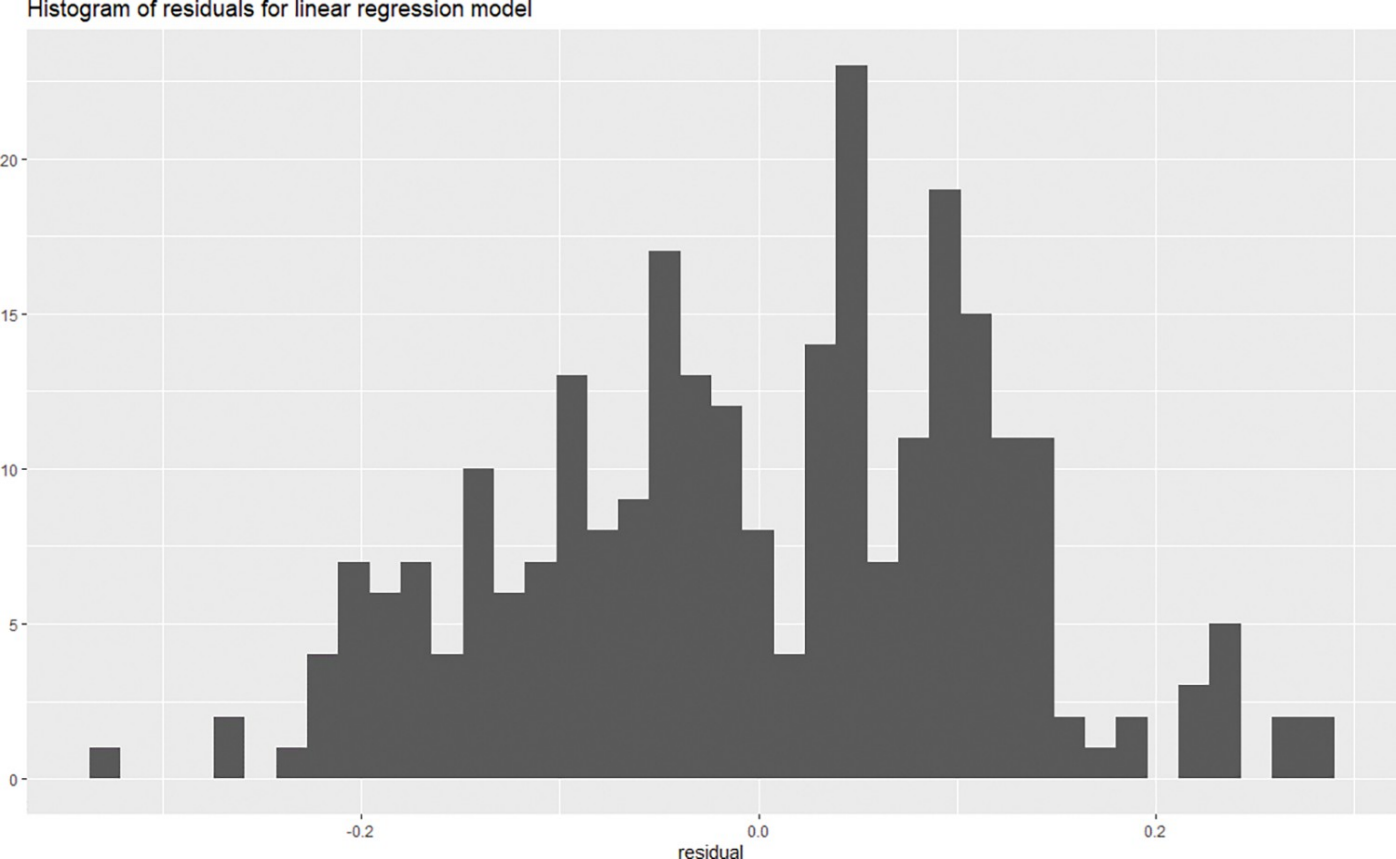
Histogram of residuals of the regression model for the effective reproduction rate.

This model has an adjusted *R*^2^ of 0.859 a corrected *R*^2^ of 0.852, and a corrected MSE of 0.014. We observe the residuals of this model to be approximately normally distributed around zero, meaning the model indicates no non-linearity (Fig 5). We check multi-collinearity of predictors by using VIF (Variance Inflation Factor). In practice, there are typically a small amount of collinearity among predictors in a regression model. Staying home behaviour and traveling behaviour have a VIF value just above 10. A VIF value of 10 is considered to be a threshold for high collinearity [45].

*Interactions and their effect on effective reproduction rate*. Additionally we determine the value of adding interaction terms to the linear model. The model with interaction terms performs well in terms of *R*^2^ and test error with an adjusted *R*^2^ of 0.889, a corrected *R*^2^ of 0.882, and a corrected MSE of 0.011. Yet interactions do not show a significant impact for the prediction of the effective reproduction rate. For this reason, we do not include interaction terms in the multiple linear regression model to not overcomplicate the model.

*Results of our regression model for effective reproduction rate*. The normalized values of coefficients of the regression model and the standard deviation of these estimates are provided in Table 2. We express the statistical significance of the relation between a parameter and response with help of the *p-*value. We consider parameters to be statistically significant when a *p*-value is below 0.01. In Table 2, “***” stands for a *p*-value <0.001 and “*” for a *p*-value <0.05.

**Table 2:** Normalized values of coefficients, standard deviation and p-value for all predictors in the regression model of the effective reproduction rate.

Predictor	Estimate	Std. Error	P-value
*Intercept*	1.333	0.141	< 2e-16 ***
*Average temperature*	-0.380	0.078	1.99e-06 ***
*Staying home behaviour*	-0.454	0.111	5.91e-05 ***
*Traveling behaviour*	0.801	0.144	5.66e-08 ***
*School closure*	0.189	0.061	0.00228 ***
*Catering services closure*	-0.162	0.084	0.05401 *
*Event allowance*	-0.303	0.073	4.04e-05 ***
*Facemasks*	0.184	0.041	1.09e-05 ***

#### 3.2.3 Regression model for the number of confirmed cases

**Fig 6:**
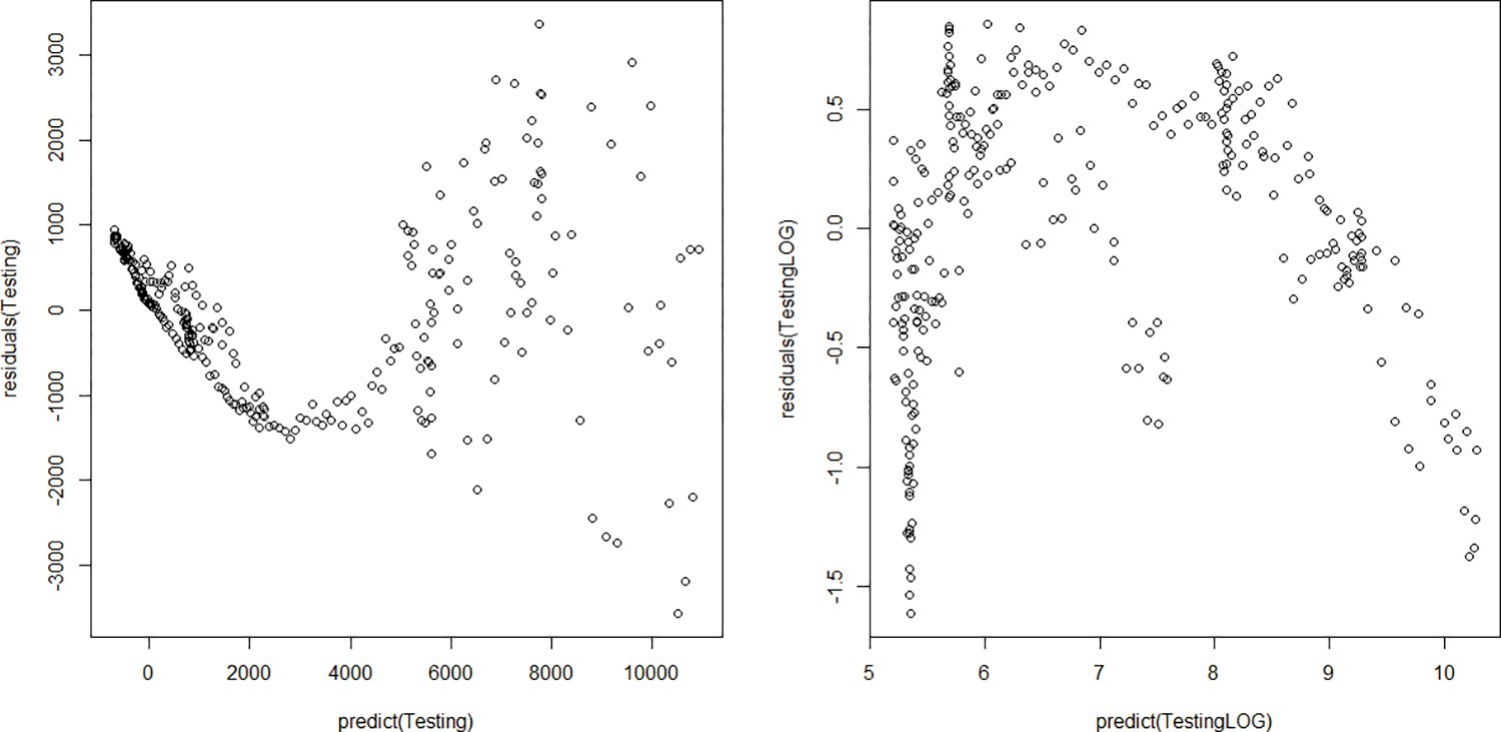
Residual plot of the regression models for the number of confirmed cases. On the left the model without logarithmic response and on the right the model with a logarithmic response.

The number of confirmed cases highly depends on the number of infections and the number of tests per day. Since the actual number of infected cases per day is unknown, this number has to be estimated. We do this with data of the number of infectious cases [46]. We use a logarithm of the response variable as we observe that the residuals are more evenly distributed in a logarithmic model (see Fig 6). As a result, we find that the relation between the number of confirmed cases per day and the predictors, number of tests per day and number of infections per day, is significant. The following expression for the number of confirmed cases on any day *t* is obtained:

$$confirmed\;cases\;(t)=e^{4.869+4.56e-05*number\;of\;tests+1.361e-04*infected\;cases}$$

In Table 3, the estimates of the coefficients *β*_*i*_ for the predictors in this model can be found, together with their *p*-value and the standard error of the estimates for *β*_*i*_.

**Table 3:** Values of coefficients, standard deviation and p-value for all parameters in the regression model of the number of confirmed cases.

Predictor	Estimate	Std. error	P-value
(Intercept)	4.869	5.434e-02	< 2e-16 ***
Number of tests	4.559e-05	2.413e-06	< 2e-16 ***
Number of infected cases	1.361e-04	6.797e-06	< 2e-16 ***

### 3.3 Calibration of the SD model

To obtain actual spread, the values of fixed parameters are calibrated (see Table 4). With a calibrated incubation period of 4 days and a calibrated infectious duration of 5.5 days respectively, we obtain an incubation rate α of 1/4 and recovery rate *γ* of 1/5.5.

**Table 4:** Calibrated input values of the fixed parameters in the SD model.

Calibrated parameter	Calibrated input value
*Incubation period*	4
*Infectious period*	5.5
*Basic reproduction rate*	2.5
*Initial infected cases*	100
*Infection fatality ratio*	0.37%
*Self-quarantine fraction*	60%

### 3.4 Modelling the policies to prevent spread

In this section we describe how each policy, doing nothing, mitigation, curbing, and elimination is modelled. In each of these policies the strictness of measures differs.

### Measures applied per policy

**Fig 7:**
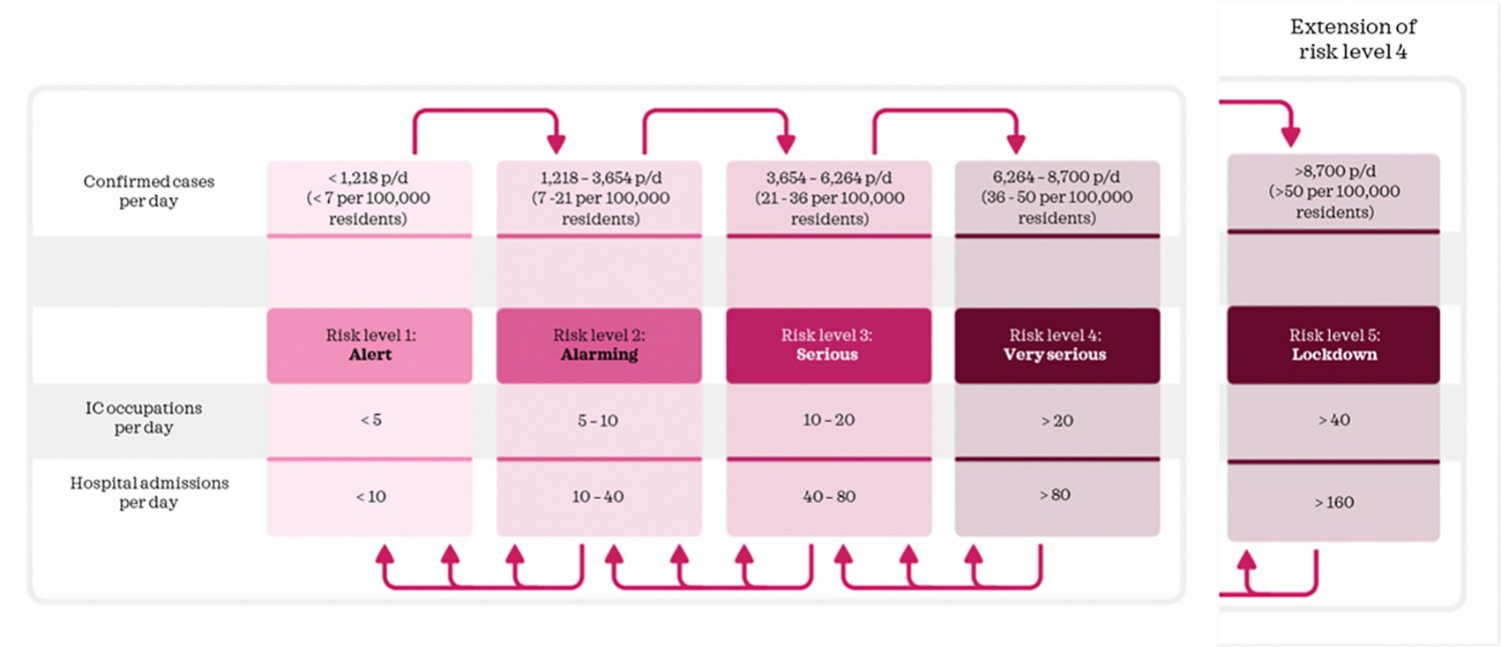
Route map to determine the risk level.

**Fig 8:**
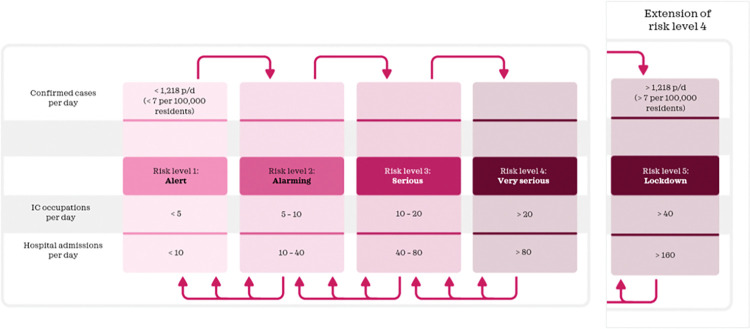
Route map to determine the risk level with adjusted signal values.

**Fig 9:**
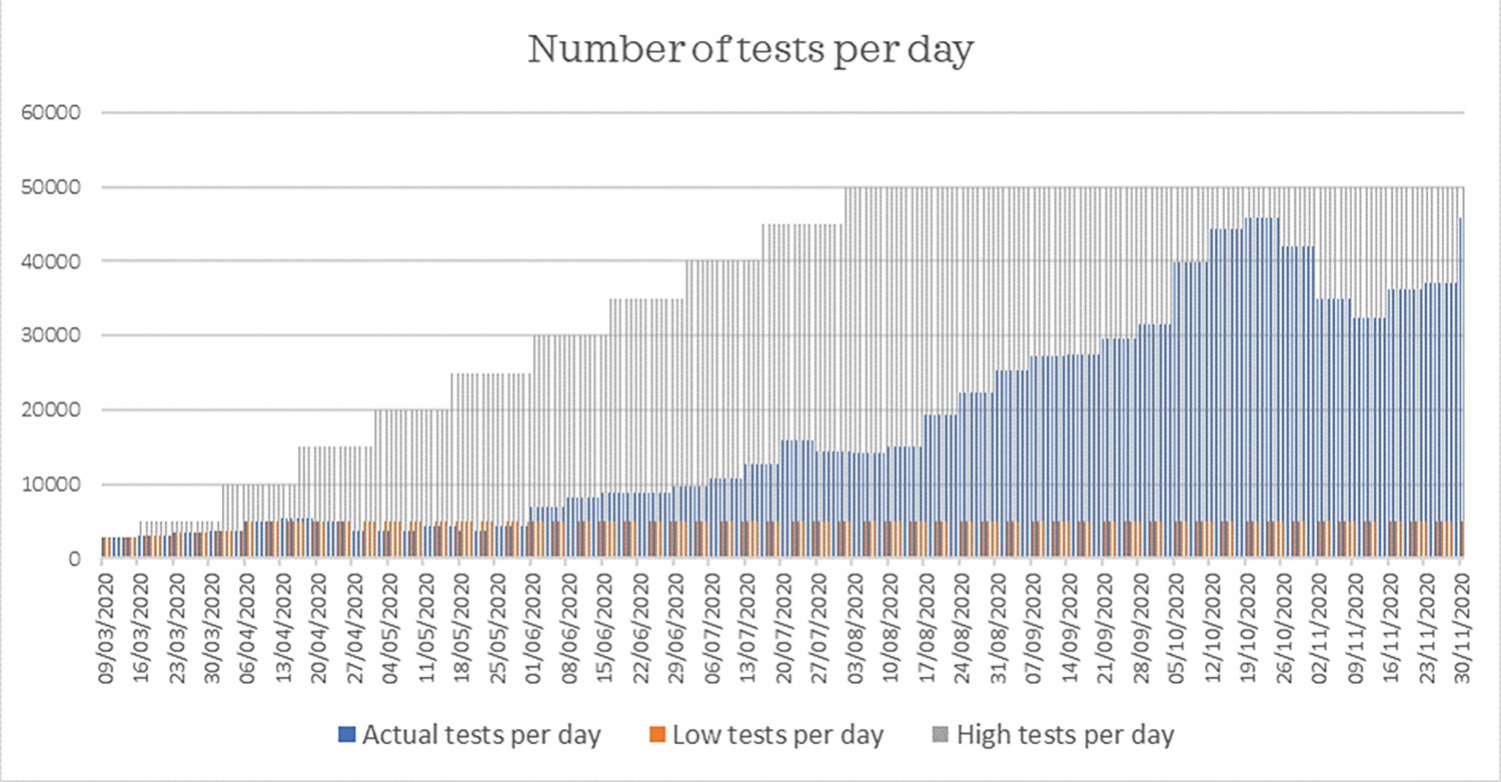
Development of low, actual and high testing capacity per day.

- *Do nothing*. No measures (Table 5) are implemented and a low testing capacity (Fig 9) is used.
- *Mitigation*. The number of IC occupations and hospital admissions on a day are used to identify the measures to implement for this policy. Signal values for IC occupations and hospital admissions per day (Fig 7) determine the risk level and subsequently the measures that are implemented (Table 5). Furthermore, the actual number of tests per day is used (Fig 9).
- *Curbing*. To determine the performance of curbing, we distinguish two ways to implement the curbing policy, referred to as curbing type 1 and curbing type 2. The first type uses signal values from the route map of the Dutch government to determine when to implement certain measures (Fig 7). The second type uses an adjusted route map (Fig 8). The number of confirmed cases is used to identify the measures to implement for the curbing policy, because it indicates a change or trend in spread earlier than other indicators such as the number of hospitalizations. And since curbing requires a high testing capacity to quickly identify infected cases, this policy uses a high testing capacity (Fig 9).
- *Elimination*. Lockdown measures apply in the entire period and testing capacity is set high to help quick tracking of infected cases.

**Table 5:** Established input values per risk level for each measure.

	No measures	Alert	Alarming	Serious	Very serious	Lockdown
*School closure*	1	2	2	3	4	5
*Catering service closure*	1	2	3	4	5	5
*Event allowance*	1	1	2	3	4	5
*Traveling behaviour*	1	0.5	0.5	0.3	0.3	0.2
*Staying home behaviour*	0	4.5%	4.5%	9%	13.5%	18%

In order to model the mitigation and curbing policies, we use the so-called route map of the Dutch government [47]. The Dutch government uses this route map to determine which measures to apply with help of signal values (Fig 7). Arrows in the figure indicate the risk level that applies when certain signal values are reached and consequently which measures have to be implemented.

In Table 5, we establish the values each measure should have per risk level (Figs 7 and 8). For school closure, catering services closure and event allowance, this is in accordance with the values per risk level in the route map of the Dutch government and parameter values in Table 1. For factors traveling behaviour and staying home behaviour the established values are approximations, based on observed values in the past (Figs 2 and 3). We assume that measures from a lower or higher risk level can only be implemented when measures of the current risk level are implemented for at least two weeks, because effects of adjusted measures are not immediately visible.

For each policy, the input values used for all measures, and the factors testing capacity, staying home behaviour, traveling behaviour, and weather related factors can be found per day in de S1 Data.

## 4.Results

The results after calibrating and validating the model are provided in Section 4.1. In Section 4.2, the results of the modelled policies (doing nothing, mitigation, curbing, and elimination) and the sensitivity results can be found.

### 4.1 Performance of the model

We express the performance of the SD model with numbers of the infected cases, confirmed cases, deaths, hospitalizations, and IC occupations.

#### 4.1.1 Calibration results

Calibrated numbers are values observed on 30 November. The calibrated results for all indicators but the total infected cases in our model are quite aligned with actual values (see Table 6).

**Table 6:** Performance of the SD model after calibration.

	Total infected	Total confirmed	Total deaths	Total hospitalizations	Total IC occupations
*Calibrated numbers*	3,013,040	528,381	9,419	27,946	5,589
*Actual numbers*	N.A.	529,304	9,653	27,738	5,551

Development of the calibrated number of infected cases, confirmed cases, deaths, hospitalizations, and IC occupations per day is provided in S2–S6 Figs.

#### 4.1.2 Validation results

Numbers from 1 December until 31 December are used to validate our model. Table 7 expresses the actual and validated numbers of the infected cases, confirmed cases, deaths, hospitalizations, and IC occupations on 31 December. Development of the infected cases, confirmed cases, deaths, hospitalizations, and IC occupations per day are provided in S9–S11 Figs.

**Table 7:** Performance of the SD model after validation.

	Total infected	Total confirmed	Total deaths	Total hospitalizations	Total IC occupations
*Validated numbers*	3,534,680	810,482	11,686	33,884	6,777
*Actual numbers*	N.A.	808,906	11,627	34,833	6,748

### 4.2 Performance of the policies

We compare the performance of policies based on two indicators: the number of infected cases and the number of days with strict measures. The first provides an indication of the spread of the virus and the second an indication of the impact on economy and social life.

#### 4.2.1 Results of the policies

In Table 8, we provide the numbers for infected cases, confirmed cases, deaths, hospitalizations, and IC occupations on 30 November per policy. We refer to the “actual policy” as the policy that leads to the numbers observed in reality. Graphical results over time of all policies are provided in S12 and S13 Figs.

**Table 8:** Performance of the policies in terms of spread.

Policy	Total infected	Total confirmed	Total deaths	Total hospitalizations	Total IC occupations
*Actual policy*	3,013,040	528,381	9,419	27,946	5,589
*Doing nothing*	11,927,500	7,962,770	44,022	117,404	23,481
*Mitigation*	2,590,550	229,439	8,797	24,678	4,936
*Curbing type 1*	4,806,090	1,113,440	16,528	46,190	9,238
*Curbing type 2*	2,193,390	332,144	8,089	21,582	4,316
*Elimination*	1,142,350	109,318	4,229	11,256	2,251

In Table 9, we provide the number of days with strict measures per policy. Strict measures are considered to be measures of the serious, very serious, or lockdown risk level. We cannot provide a clear specification of the days with strict measures for the actual policy, because in reality the Dutch government did not determine the policy exactly according to the signal values in the route map.

**Table 9:** Performance of the policies in terms of days with strict measures.

Policy	Days with strict measures
Actual policy	+/- 200
Doing nothing	0
Mitigation	260
Curbing type 1	131
Curbing type 2	91
Elimination	264

#### 4.2.2 Sensitivity results of feasible policies

The SD model, being a nonlinear model, can be very sensitive to small changes. For this reason, we simulate the policies again considering small changes in input values of the self-quarantine fraction and the testing capacity. These two parameters are considered to be the main parameters that can be affected in reality. We let the self-quarantine fraction vary between 20%, 60% and 80% and let the testing capacity vary between low and high.

**Fig 10:**
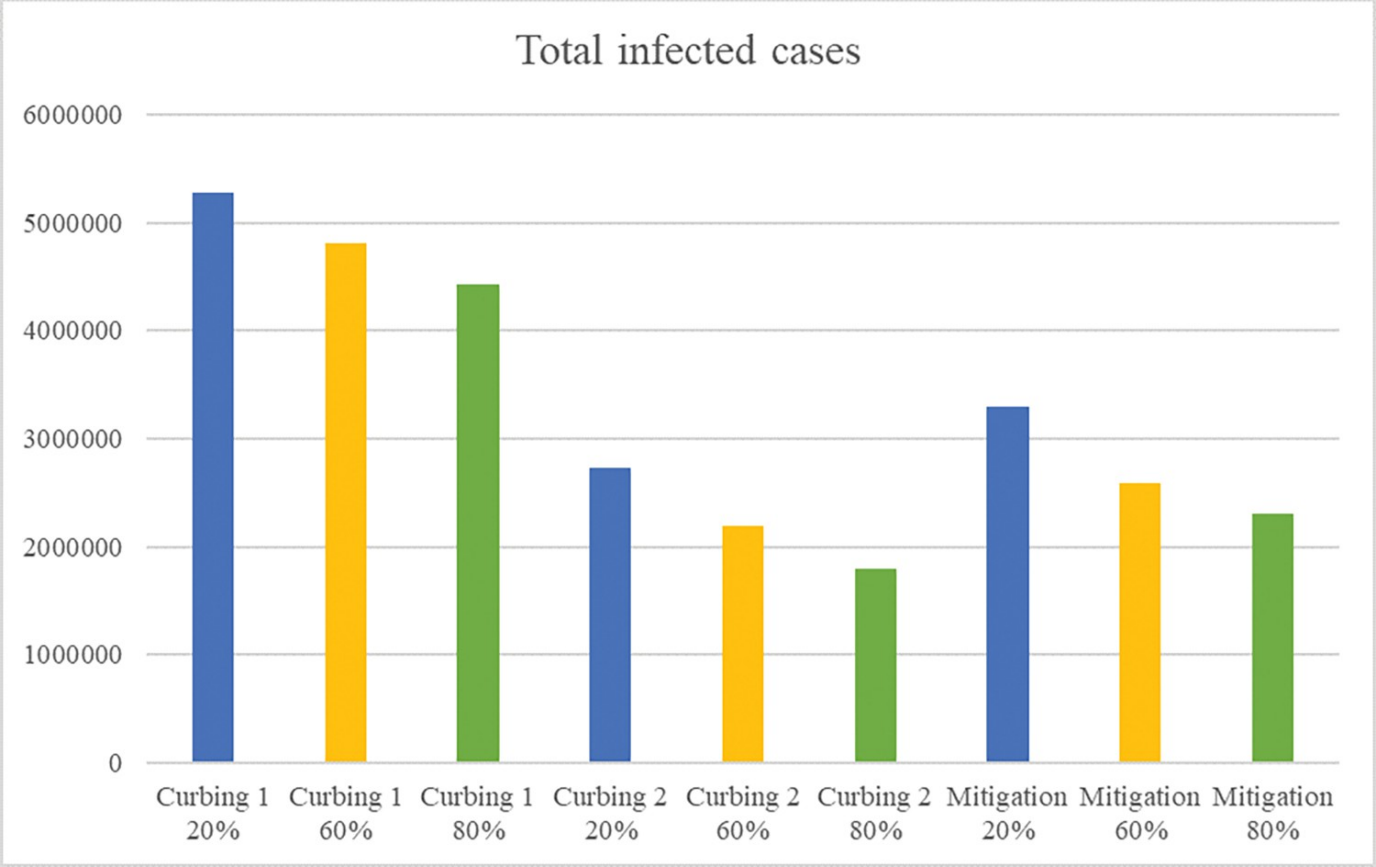
Sensitivity results of the total infected cases on 30 November 2020 per policy–changing quarantine fraction.

**Fig 11:**
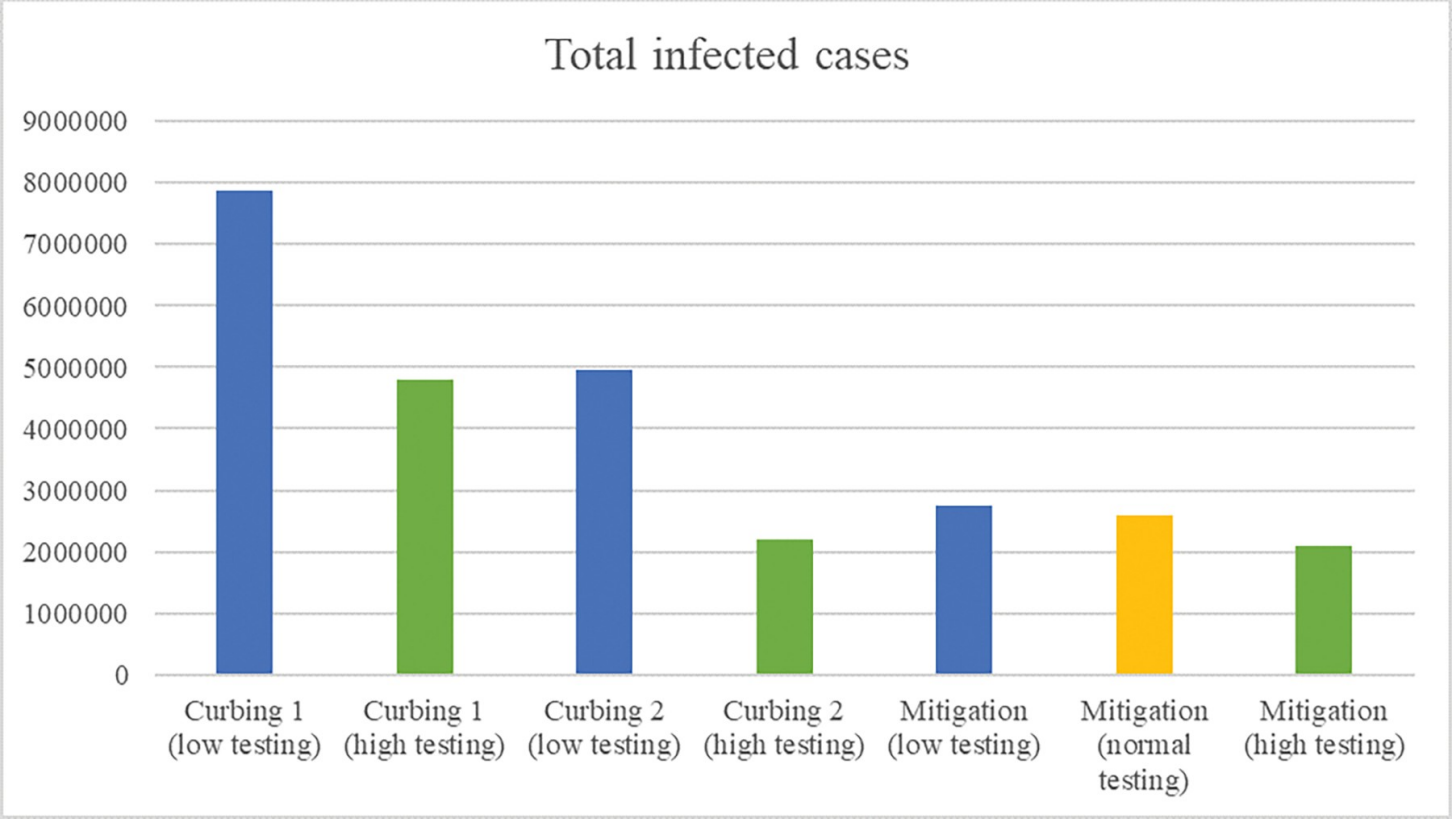
Sensitivity results of the total infected cases on 30 November 2020 per policy–changing testing capacity.

Only feasible policies are simulated with these varying input values. As feasible policies we consider mitigation and curbing (type 1 and type 2). Results for the number of infected cases on 30 November are provided in Figs 10 and 11 and results for the number of days with strict measures in Tables 10 and 11.

**Table 10:** Sensitivity results of the number of days with strict measures per policy–changing quarantine fraction.

Policy	Days with strict measures
Actual policy	+/- 200
Mitigation (60%)	260
Mitigation (20%)	260
Mitigation (80%)	260
Curbing type 1 (60%)	131
Curbing type 1 (20%)	218
Curbing type 1 (80%)	113
Curbing type 2 (60%)	91
Curbing type 2 (20%)	162
Curbing type 2 (80%)	84

**Table 11:** Sensitivity results of the number of days with strict measures per policy–changing testing capacity.

Policy	Days with strict measures
Actual policy	+/- 200
Mitigation (low testing capacity)	260
Mitigation (normal testing capacity)	260
Mitigation (high testing capacity)	239
Curbing type 1 (low testing capacity)	97
Curbing type 1 (high testing capacity)	131
Curbing type 2 (low testing capacity)	126
Curbing type 2 (high testing capacity)	91

## 5. Discussion and conclusions

### 5.1 Discussion

Doing nothing to prevent spread of COVID-19 turns out to be the worst policy in the Dutch case. This is expected due to the high population density in the Netherlands. The feasibility of the elimination policy is low in a short amount of time, since it requires world-wide cooperation. There are some countries that tried to implement this policy, for example New Zealand, resulting in very low infection numbers combined with a low number of days with strict measures [48]. This might partially be achieved due to a lower population density in these countries. We believe that, with the relatively high population density in the Netherlands, the elimination policy would cause major damage to economy and is therefore not feasible in the Dutch case. We see this in economic performance during periods of lockdown measures [49].

Mitigation turns out to be the policy that comes closest to the actual situation in the Netherlands. This is expected because the actually implemented policy resembles this policy the most. The total number of infected cases with the mitigation policy is lower than the actual number (2,590,550 versus 3,013,040), yet the number of days with strict measures for the mitigation policy is approximately 60 days higher. This indicates that the signal values in the route map of the government are not sufficient to come from a higher risk level to a lower risk level quickly, leading to a lot of days with strict measures and consequently a bigger impact on economy.

With the curbing policy, infected individuals will be identified by quick testing and in-depth source- and contact investigation. We studied two types of curbing by which we observe that curbing becomes more effective as it has stricter measures. Looking at the differences between the two types of curbing we observe that implementing stricter short-term measures are more effective than gradual long-term measures. This is more resilient for economy as it will lead to lower negative impacts. Especially, if such a policy can be announced to public, the public and businesses can plan according to such clear plans and this can reduce sources of uncertainties in the public and business.

An important requirement of the curbing type 2 policy to be effective is having a high testing capacity. The sensitivity results show that when mitigation uses a high testing capacity, the total number of infected cases becomes lower than the number of infected cases of curbing type 2 (2,097,410 versus 2,193,390). With a remark that the number of days with strict measures is more than once as high (239 versus 91). When both policies implement a low testing capacity, the number of infected cases of mitigation is significantly lower than the number infected cases of curbing type 2 (2,763,400 versus 4,969,410), where the number of days with strict measures remains more than once as high (260 versus 126). Similar to testing capacity, effective self-quarantine has a significant effect on the spread of the virus and the impact on economy. Based on the results in the sensitivity analysis, both the number of infected cases and the number of days with strict measures decrease significantly with a higher self-quarantine fraction, no matter the implemented policy. This suggests that it is valuable to invest in an effective testing and quarantine policy, which is in line with other works [19, 50].

Another finding of our model is that the actual total number of infected cases seems to be highly underestimated when we compare it to the estimate of the number of infected cases. The total number of infected cases based on this estimate is 1,814,638 on 30 November, whereas the calibrated total number of infected cases in our model is much higher (3,013,040).

The outcome of this study can be different for other countries due to the high population density in the Netherlands. However, our model could still be applicable to other countries. This requires adjustments of the factors and the government measures.

### 5.2 Limitations

To express the measures school closure, caterings services closure, event allowance, and wearing facemasks we use discrete values (levels 1 to 5). By using continuous values we can obtain a better estimation of *R*_*e*_^*linear*^(*t*), which can improve predictions.

Not all measures that are included in the route map of the Dutch government are included in our analysis (e.g. measures on sports and the curfew). The measures that we do not include might show their impact in the regression model for the effective reproduction rate with another measure that is included.

Our paper focuses on the early phase of the pandemic, before the introduction of vaccines. The variants of SARS-CoV-2 and the vaccination have a huge impact on the spread of the pandemic. Extension of our model to include the variants and vaccination would be worth to investigate.

### 5.3 Conclusions

To effectively prevent the spread of COVID-19 in the Netherlands, strict measures have to be implemented early to keep both the number of days with strict measures and the number of infected cases relatively low. It is however important to combine this way of policymaking with effective self-quarantine and an effective testing policy. Our results are valid for the Dutch case. However, our model is generic and can be applicable for other cases provided that the data is available. As we focus on the early of the pandemic, we exclude variants of SARS-CoV-2 and vaccination. These are the notable directions that deserve further attention.

## Supporting information

S1 Table Historical values of the measures with additional dates of implementation. (TIF)

S2 Table The parameters that are included in the backward regression. The linear relation between *R*_*e*_(*t*) and these parameters.
(TIF)

S1 Fig Visualization of *R*_*e*_^*calculated*^(*t*) from 20 February until 31 November, based on the number of infections and the number of hospitalizations respectively. The figure on the left is of the number of confirmed cases. The figure on the right is of the number of hospital admissions.
(TIF)

S2 Fig Calibration results of the development of the calibrated number of confirmed cases per day. (TIF)

S3 Fig Calibration results of the development of the calibrated number of infected cases per day. (TIF)

S4 Fig Calibration results of the development of the calibrated number of hospital admissions per day. (TIF)

S5 Fig Calibration results of the development of the calibrated number of IC admissions per day. (TIF)

S6 Fig Calibration results of the development of the calibrated number of deaths per day. (TIF)

S7 Fig Validation results of the number of confirmed cases per day. (TIF)

S8 Fig Validation results of the number of infected cases per day. (TIF)

S9 Fig Validation results of the number of hospital admissions per day. (TIF)

S10 Fig Validation results of the number of IC admissions per day. (TIF)

S11 Fig Validation results of the number of deaths per day. (TIF)

S12 Fig Graphical results of the total number of confirmed cases of all policies. (TIF)

S13 Fig Graphical results of the total number of infected cases of all policies. (TIF)

S1 Data (ZIP)
