# Supplementary figures and images for: Predicting the spread of covid-19 and the impact of government measures at the early stage of the pandemic: The Dutch case—Stricter but short-term measures are better
Source: PLoS One. 2023 May 12;18(5):e0283086. doi: 10.1371/journal.pone.0283086 (PMC10180597; doi:10.1371/journal.pone.0283086)

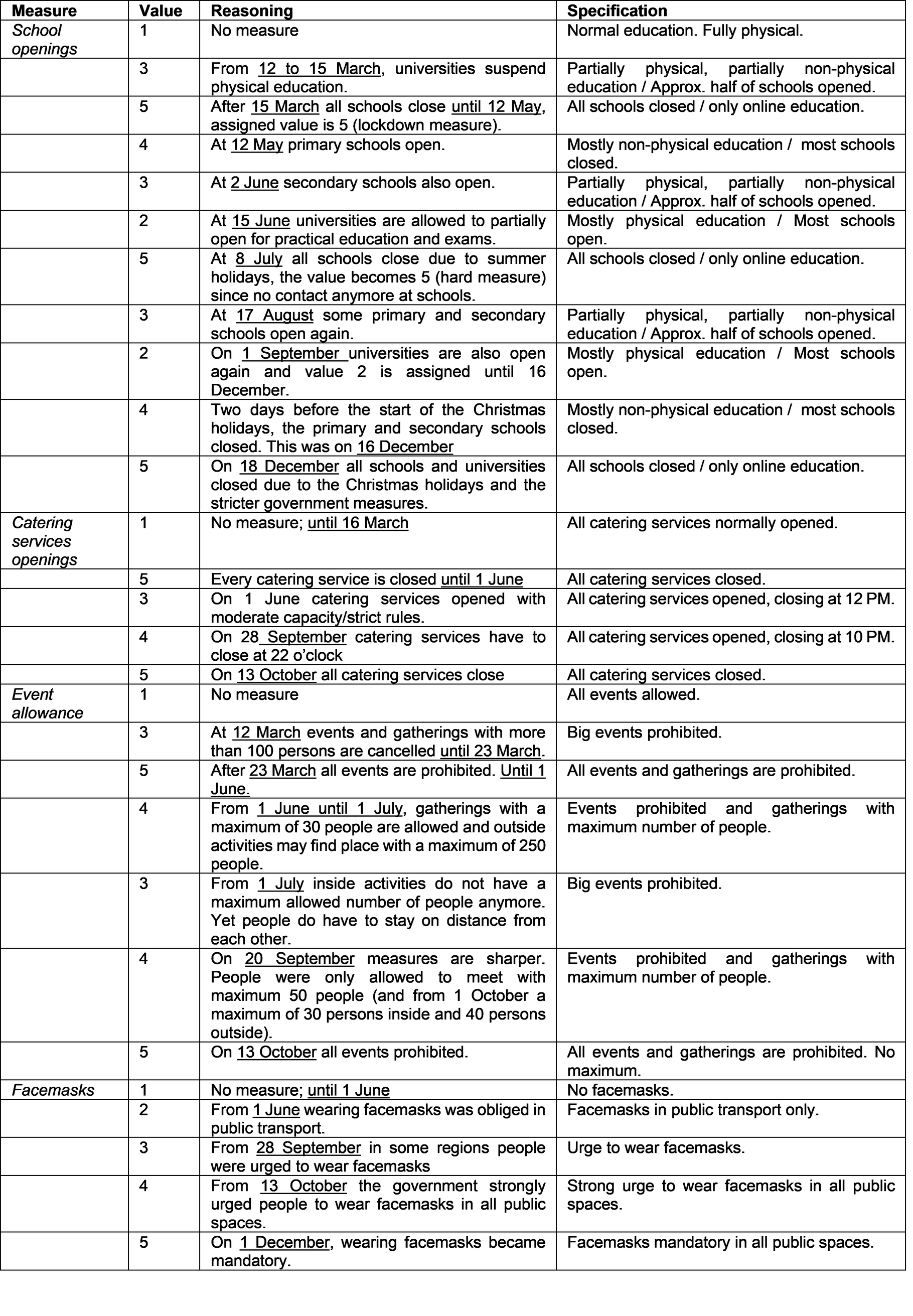

Supplement: S1 Table — (TIF) [file pone.0283086.s001.tif]

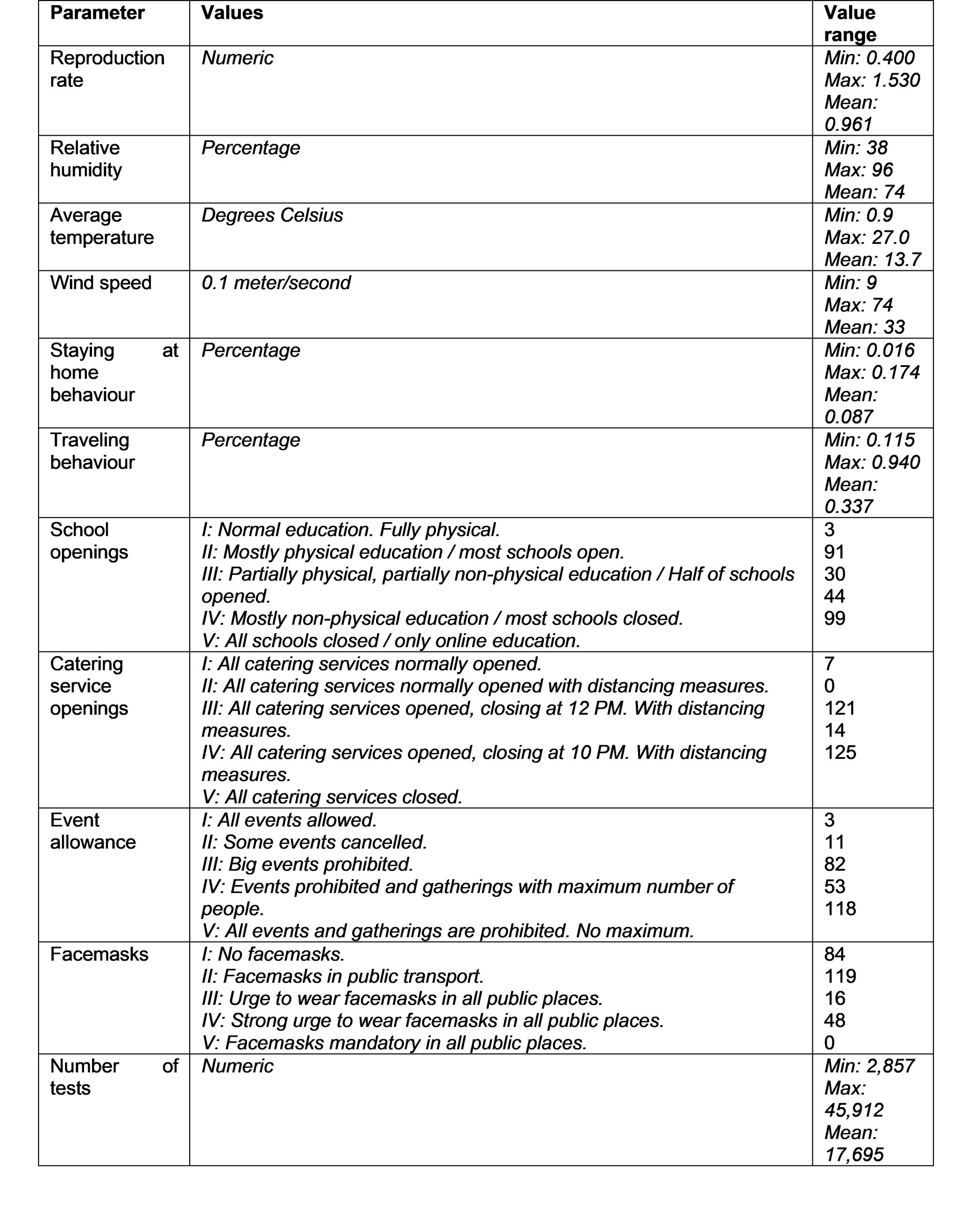

Supplement: S2 Table — The linear relation between Re(t) and these parameters. (TIF) [file pone.0283086.s002.tif]

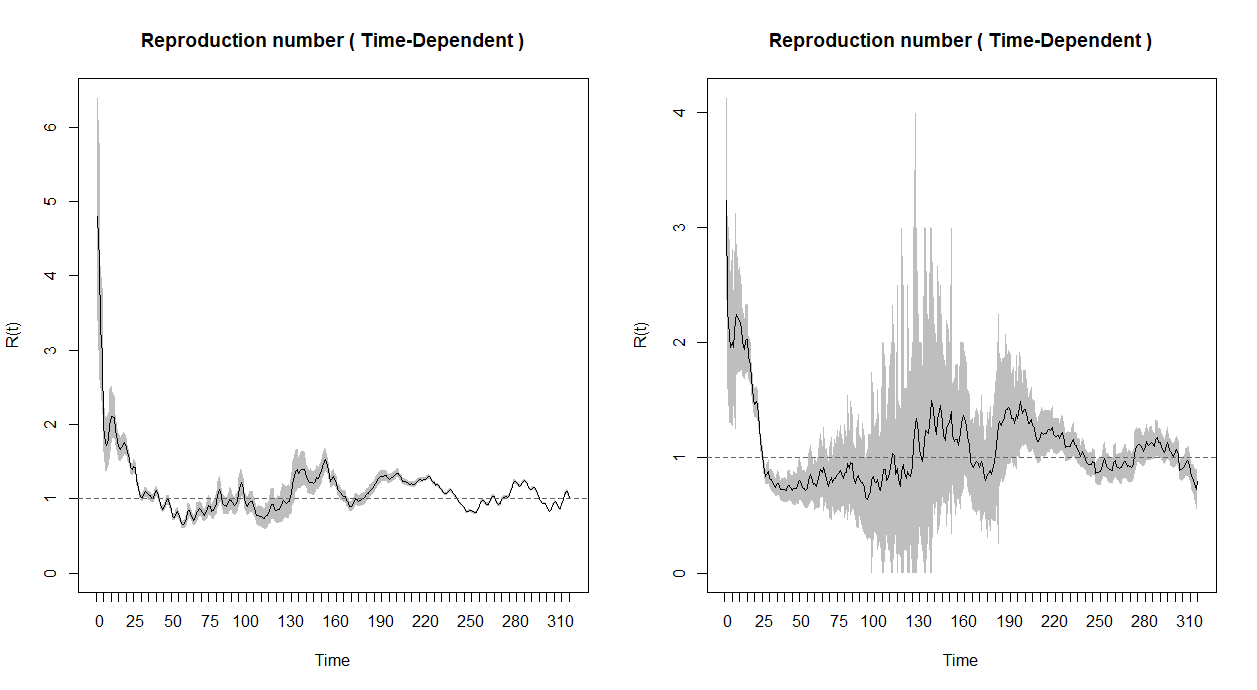

Supplement: S1 Fig — The figure on the left is of the number of confirmed cases. The figure on the right is of the number of hospital admissions. (TIF) [file pone.0283086.s003.tif]

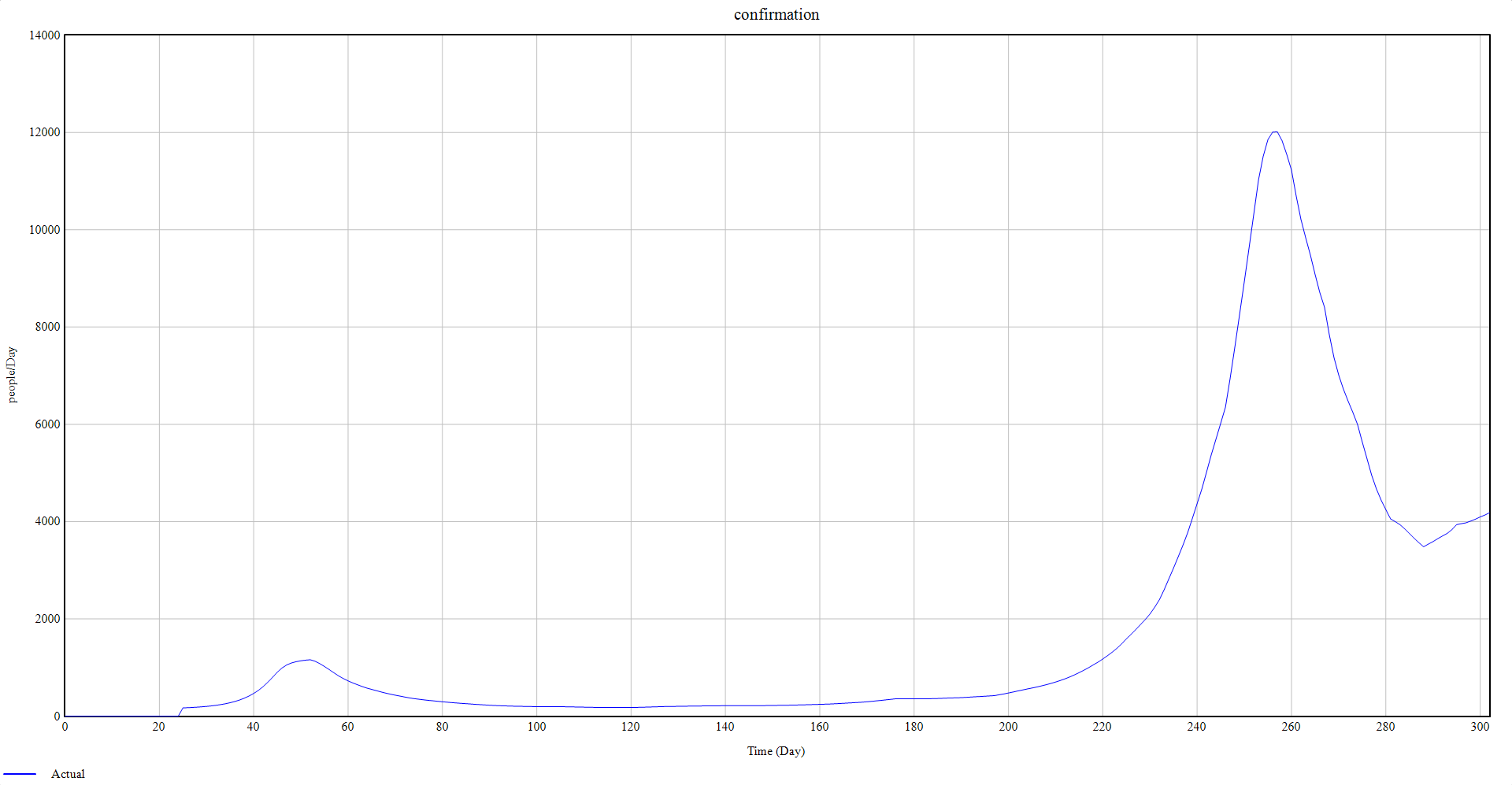

Supplement: S2 Fig — (TIF) [file pone.0283086.s004.tif]

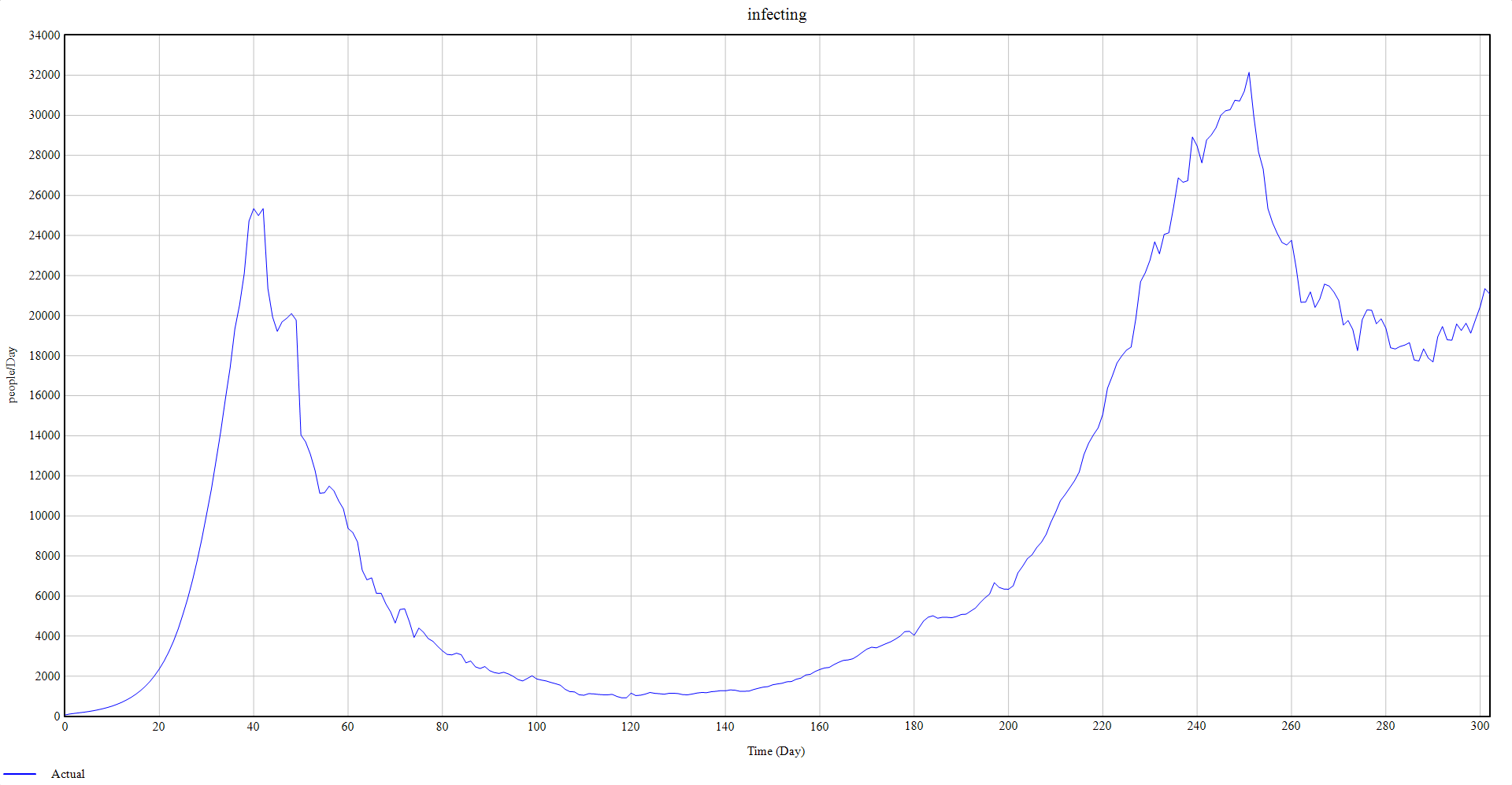

Supplement: S3 Fig — (TIF) [file pone.0283086.s005.tif]

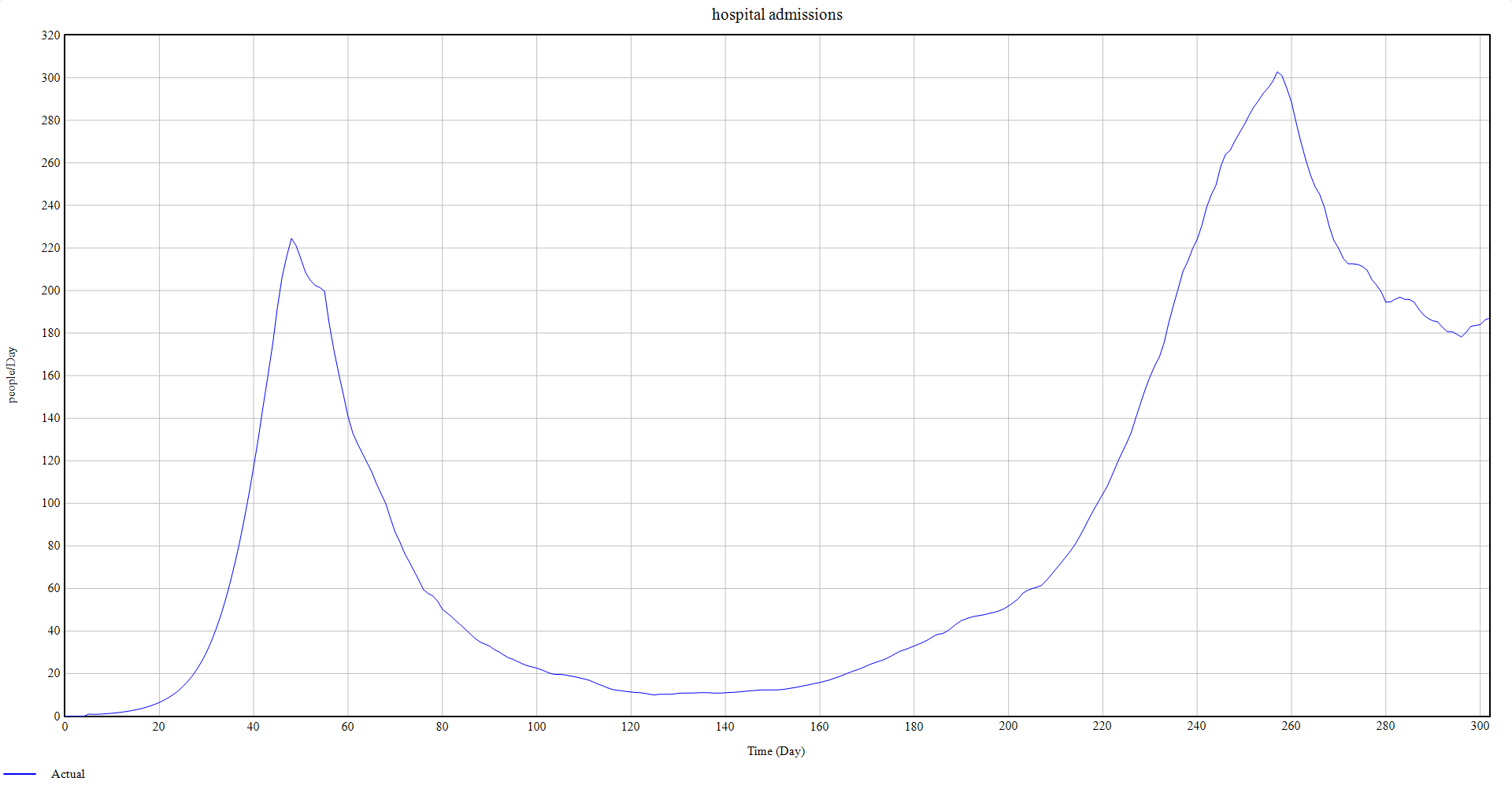

Supplement: S4 Fig — (TIF) [file pone.0283086.s006.tif]

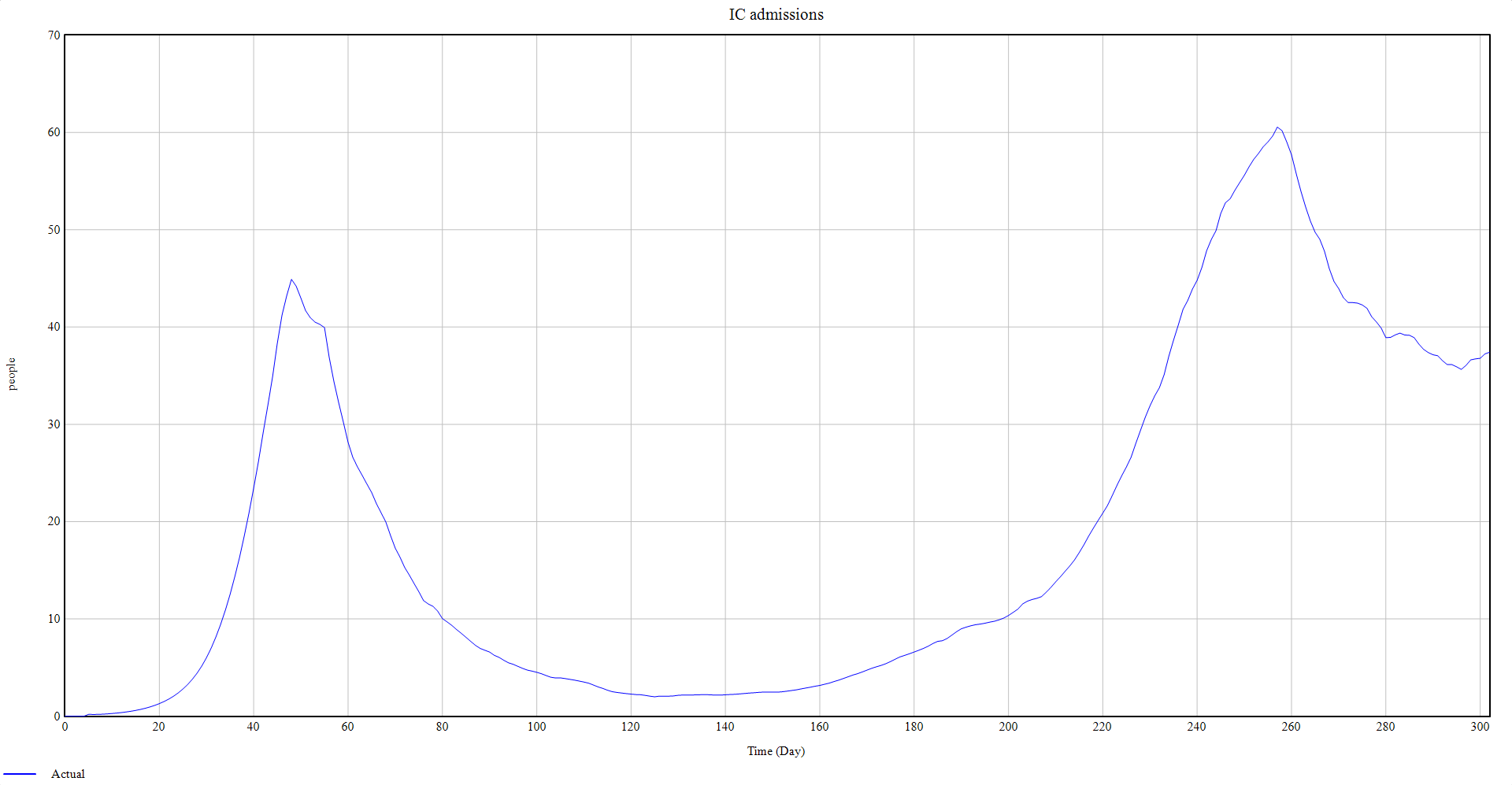

Supplement: S5 Fig — (TIF) [file pone.0283086.s007.tif]

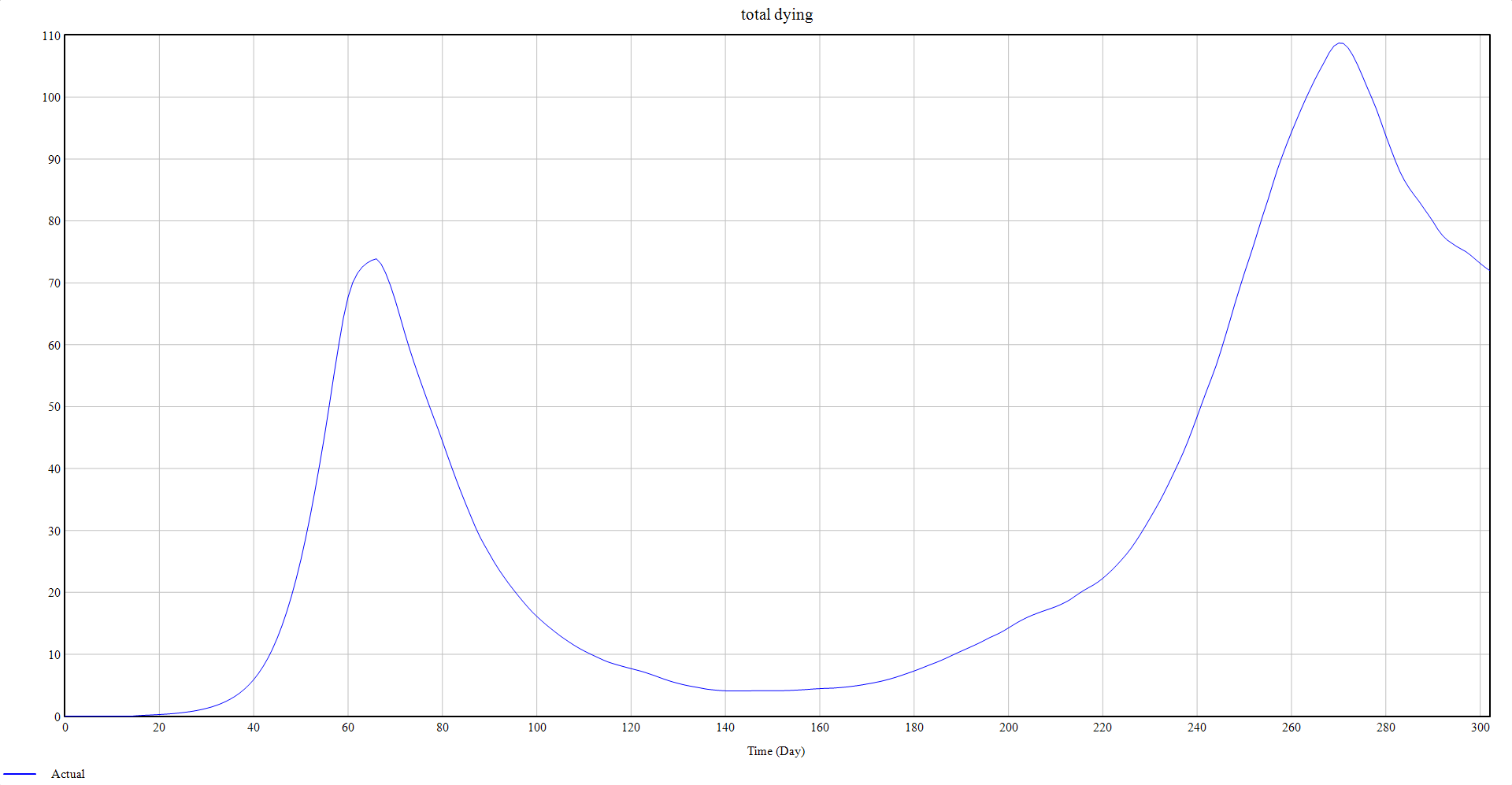

Supplement: S6 Fig — (TIF) [file pone.0283086.s008.tif]

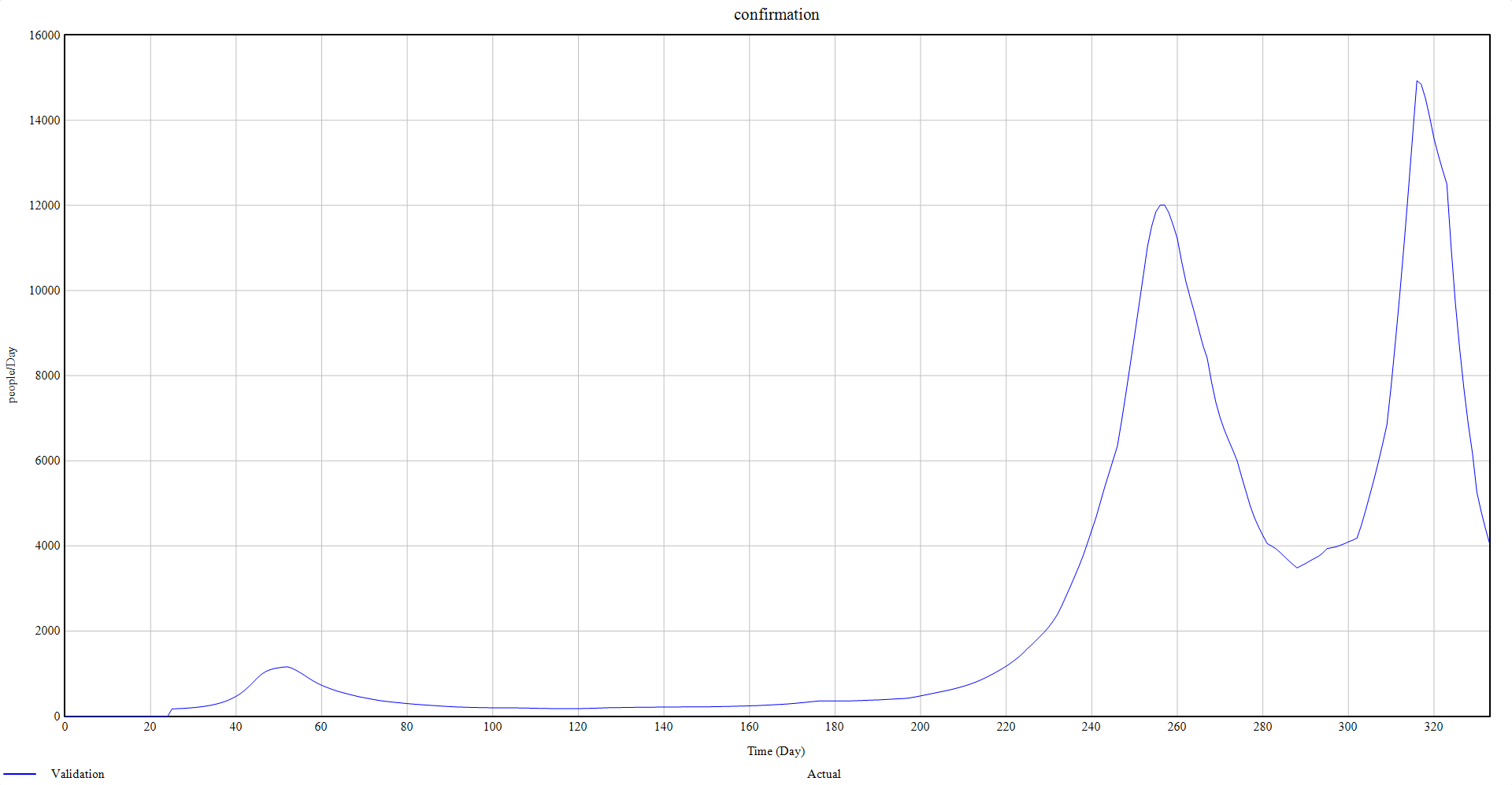

Supplement: S7 Fig — (TIF) [file pone.0283086.s009.tif]

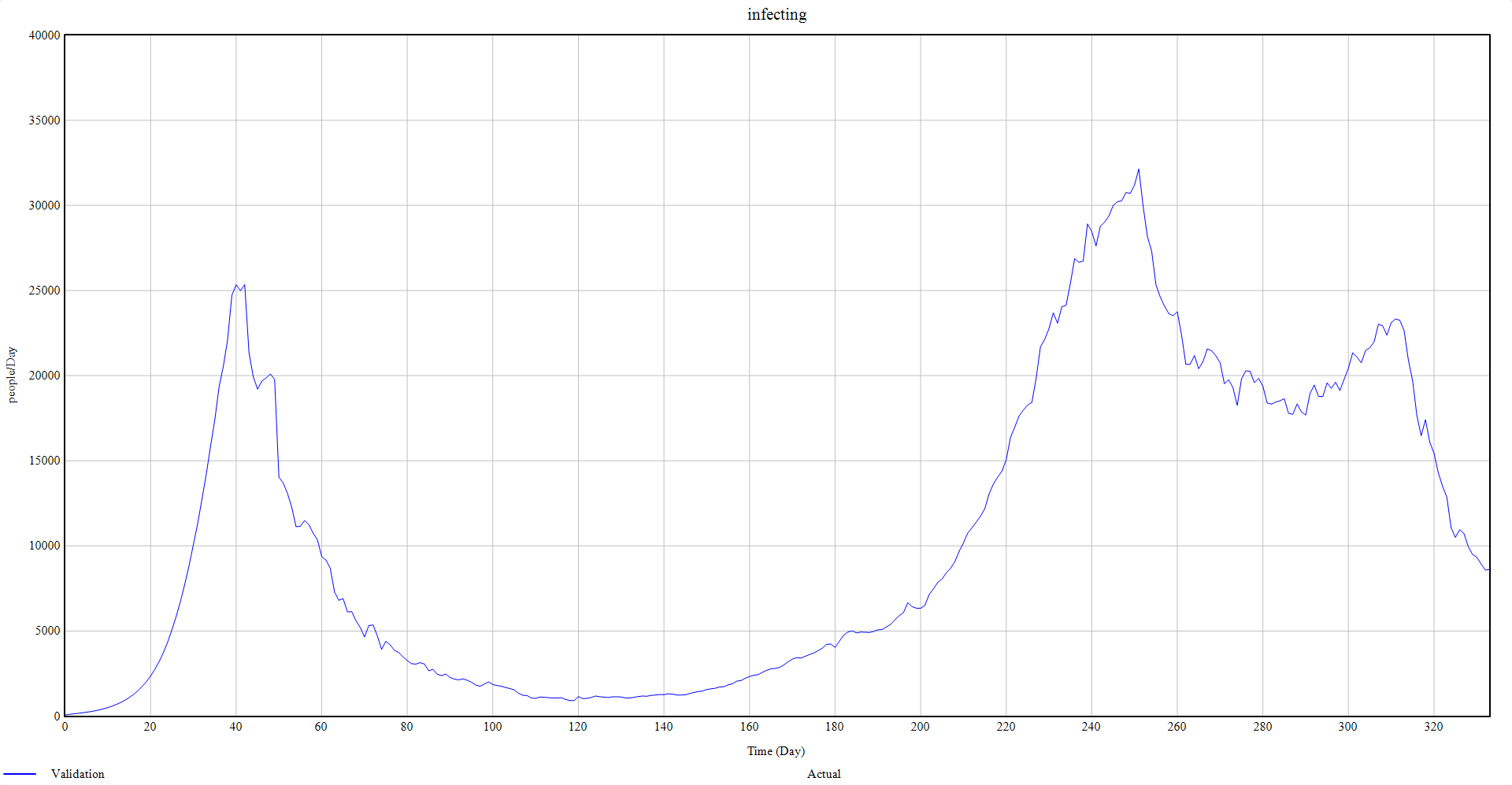

Supplement: S8 Fig — (TIF) [file pone.0283086.s010.tif]

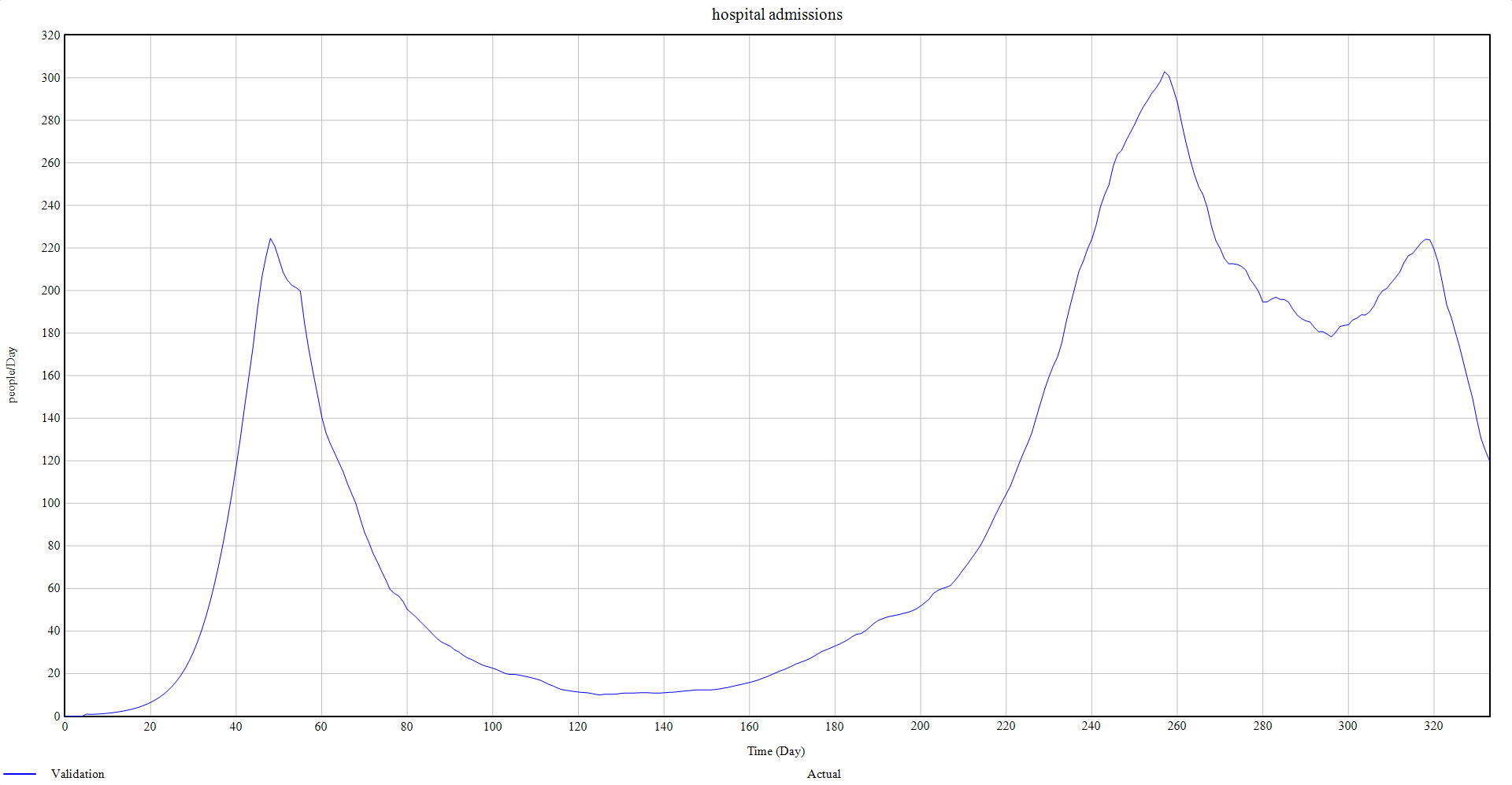

Supplement: S9 Fig — (TIF) [file pone.0283086.s011.tif]

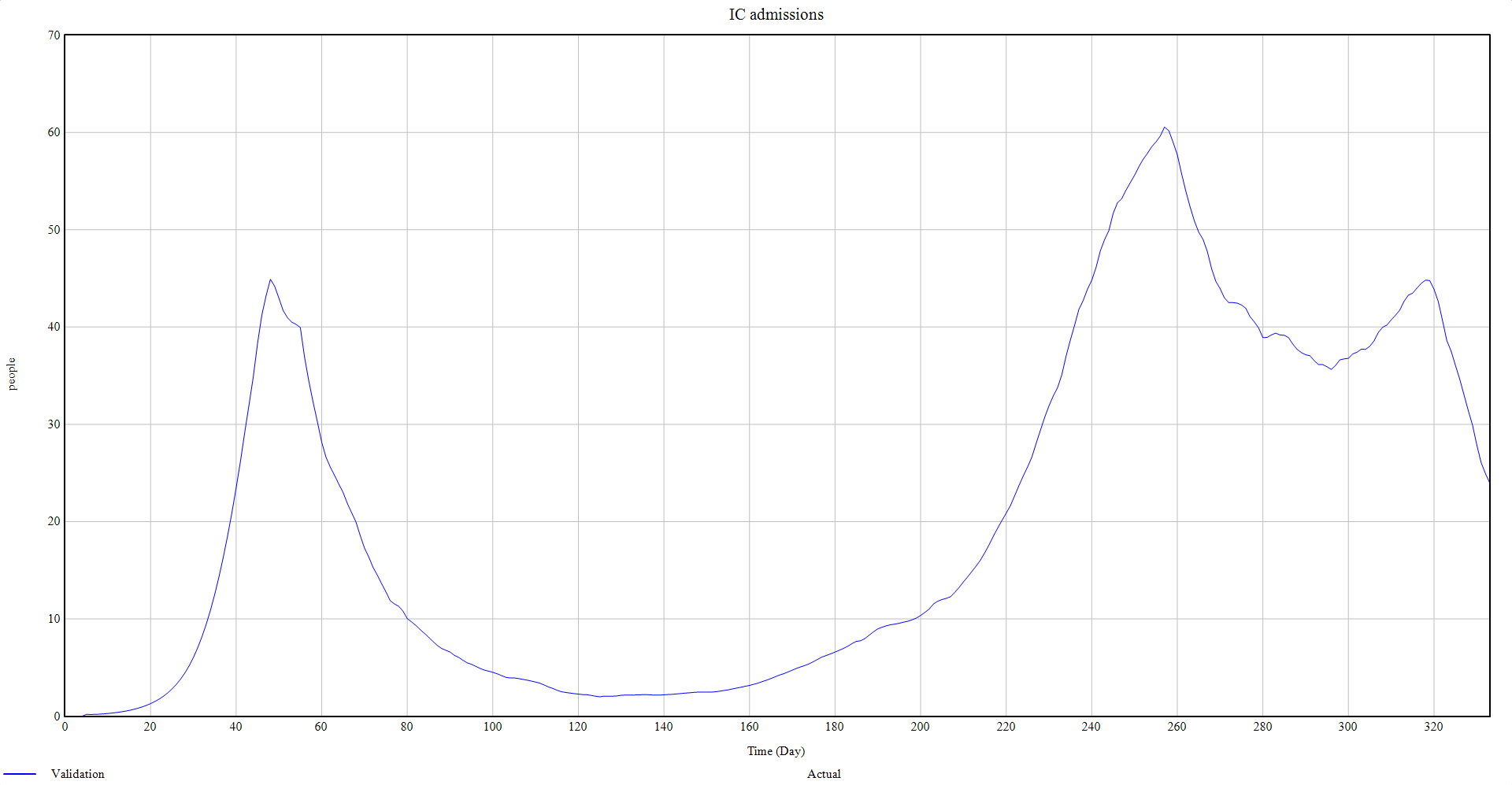

Supplement: S10 Fig — (TIF) [file pone.0283086.s012.tif]

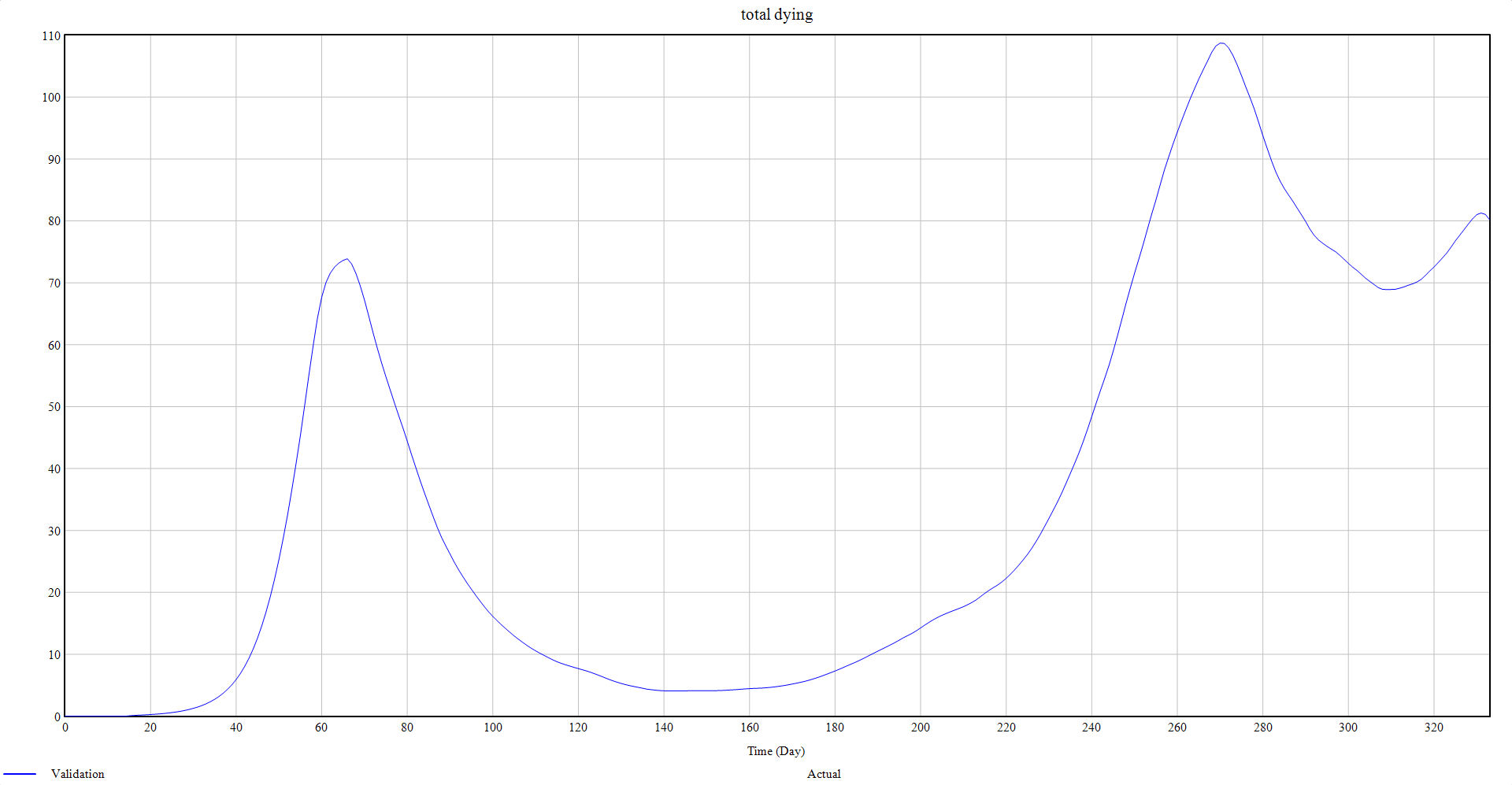

Supplement: S11 Fig — (TIF) [file pone.0283086.s013.tif]

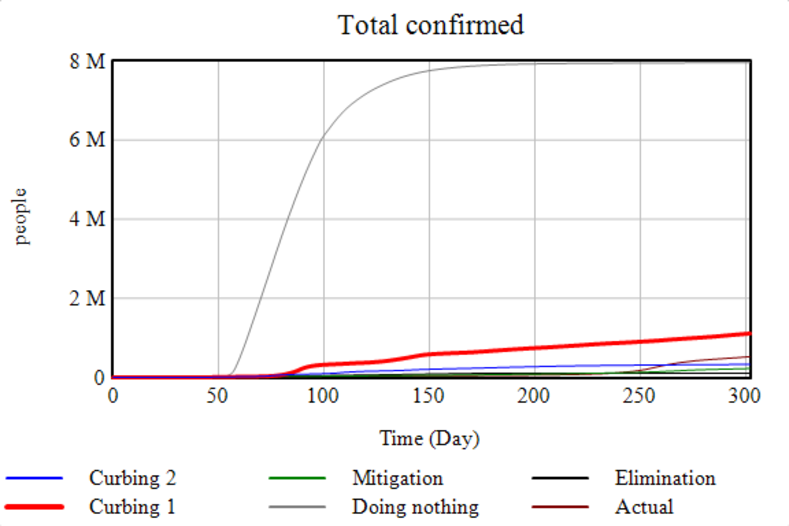

Supplement: S12 Fig — (TIF) [file pone.0283086.s014.tif]

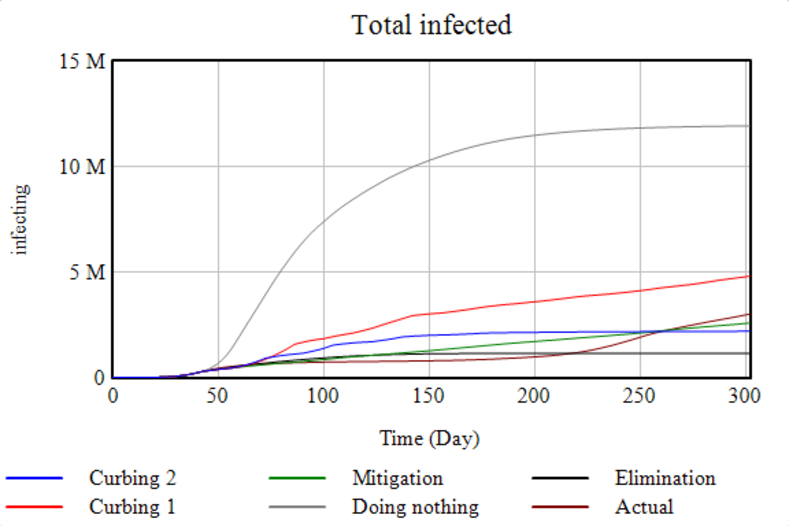

Supplement: S13 Fig — (TIF) [file pone.0283086.s015.tif]
